# Rhizosphere microbiome engineering with PGPR to combat soil-mediated climate change

**DOI:** 10.3389/fmicb.2026.1880722

**Published:** 2026-09-09

**Authors:** Noel Biju Longhinos, Pala Mahesh, Divya Vijayakumar Bindhu, Arsha Sunil, Ananya Nair, Deeksi Renukadevi Kuppusamy, Akshaya Madurai Hariharan, Vuppala Sruthi, Bipin Gopalakrishnan Nair, Sanjay Pal, Suja Subhash

**Affiliations:** Amrita School of Biotechnology, Amrita Vishwa Vidyapeetham, Kollam, India

**Keywords:** abiotic stress, climate-smart agriculture, plant growth-promoting rhizobacteria (PGPR), soil health, sustainable farming

## Abstract

The synergistic effects of accelerated climate change and anthropogenic land-use shifts increasingly compromise the functional integrity of terrestrial ecosystems. To preserve soil health and ensure global food security, a transition toward biointensive, climate-smart agriculture is imperative. This review provides a comprehensive synthesis of the multifaceted role of plant growth-promoting rhizobacteria (PGPR) as “rhizosphere architects,” bridging global biogeochemical cycles with intricate molecular and digital interventions. Microbe mediated mitigation of atmospheric stressors is evaluated by monitoring increases in soil carbon sequestration and concomitant reductions in greenhouse gas (GHG) emissions. Central to ecosystem recovery is the physiochemical regeneration of marginal and polluted soils, where PGPR facilitate the detoxification of xenobiotics, including heavy metals, microplastics, and organic contaminants, while promoting the structural restoration of degraded edaphic environments. At the tripartite plant-microbe-soil interface, the synergistic regulation of root system architecture (RSA) is investigated alongside the emerging role of epigenetic modifications, such as DNA methylation and histone acetylation, as critical drivers of transgenerational stress memory against extreme climatic conditions. These molecular and structural shifts are functionally correlated with rhizosphere enzymatic activity, including specific enzyme fluxes (e.g., urease, phosphatase, and dehydrogenase), which serve as biochemical proxies for soil health restoration. To address the historical inconsistency of field inoculants, a translational framework is proposed that integrates Industry 4.0 technologies, such as AI-assisted bioformulation design and IoT-based precision monitoring, for real-time microbiome management. By discussing the translational hurdles of microbial competition and regulatory frameworks, this synthesis underscores how the fusion of microbial ecology and digital agriculture provides a robust pathway toward resilient terrestrial ecosystems and sustained agricultural productivity in the Anthropocene.

## 1 Introduction

The current phase of global environmental change is marked by two crises: a rapidly destabilizing climate and a shrinking terrestrial resource base. Anthropogenic land-use intensification continues to threaten the functional integrity of soil ecosystems at alarming rates (Lobell et al., 2011). Environmental stresses such as prolonged drought, hypersalinity, and thermal extremes are occurring with increasing frequency and intensity and constitute a direct threat to global food security. Traditional agriculture used synthetic agrochemicals to increase yields dramatically in the past decades. But now we have reached the point of diminishing returns in our reliance on chemicals. This amplification leads to massive soil degradation and loss of microbial diversity that often exacerbates the environmental stressors it aims to mitigate. A paradigm shift away from high-input industrial agriculture is urgently needed in order to maintain agricultural output and to restore edaphic health. Thus, it is important to promote biointensive, climate-smart, and sustainable paradigms (dos Santos et al., 2025). This biological breakthrough ushers in a new era in precision microbiome management, where the deliberate use of microbial strategies is used to increase crop resilience and reduce environmental footprints.

Plant cellular functions are differently modified under abiotic stress conditions (Wang et al., 2024). Under these conditions, highly soluble, organic, electrically neutral, and nontoxic compounds with low molecular weight are accumulated as osmoprotectants or osmolytes (Slama et al., 2015) and are important in the protective role against cellular machinery damage in response to a stressful environment (Table 1). Plants naturally associate with diverse microbial communities, collectively known as the “phytomicrobiome,” which serve as an “extended functional genome” for the host (Shah et al., 2021). Rhizosphere-associated microbes are particularly critical, as they mediate rhizosphere competence and modulate plant development through intricate biochemical signaling (Orozco-Mosqueda Ma del et al., 2023). Under this paradigm, PGPR exemplify key biological assets (Ferreira et al., 2025; Sarkar et al., 2018). They also enhance plant vigor by facilitating nutrient mobilization, including diazotrophic nitrogen (N_2_)-fixation, phosphate solubilization, and siderophore-mediated iron acquisition while producing vital phytohormones like indole-3-acetic acid (IAA) (Ullah et al., 2025). Bacteria belonging to the genera *Pseudomonas, Bacillus, Azospirillum, Arthrobacter, Agrobacterium*, and *Flavobacterium* promote plant growth by facilitating carbon sequestration and the attenuation of greenhouse gas (GHG) emissions by optimizing microbial metabolic dynamics and stabilizing soil organic matter (Chandran et al., 2021). Beyond individual plant fitness, these interactions are fundamental to enhanced soil health. A core trajectory in contemporary rhizosphere research is the evaluation of PGPR in the physicochemical regeneration of marginal soils. Through the detoxification of xenobiotics and the secretion of extracellular polysaccharides (EPS), PGPR facilitate the structural restoration of degraded edaphic environments (Janssens et al., 2020; dos Santos et al., 2025). At the molecular level, these interactions induce profound epigenetic modifications, specifically DNA methylation and histone acetylation. These chromatin-level alterations represent a sophisticated mechanism of transgenerational “stress memory,” priming the host plant for sustained osmotic and oxidative resilience (Verma et al., 2024). Such molecular shifts are functionally mirrored by enhanced rhizosphere enzymatic activity, in which the flux of soil hydrolases and oxidoreductases serves as a sensitive proxy for successful ecosystem restoration (Luqman et al., 2025).

**Table 1:** Plant growth regulators involved in stress mitigation.

S. No	Plant growth regulators	Abiotic stresses	Activity	References
1	Abscisic acid (ABA)	Drought	Regulate seed maturation and dormancy, control leaf expansion and stomatal dynamics for water management, activate biochemical defense including synthesis of proline, antioxidants, LEA proteins, dehydrins, and other protective proteins, strengthens cuticular waxes to mitigate the effects of stresses.	Das et al., 2025
2	Auxin (IAA)	Drought, salinity, temperature, heavy metal stresses	Increases root and shoot growth of plants, maintains relative water content and total chlorophyll content under drought, mitigates heat stress during pollen germination.	Jing et al., 2023
3	Cytokinin (CK)	Drought, salinity	CKs support normal growth and development under stress. Enhanced photosynthetic growth leads to improved crop yield and plant health, also improves plant immunity against phytopathogens.	Giri and Virk, 2025
4	Ethylene (ET)	heat, drought, chilling salinity, heavy metals, flood	Regulates leaf senescence and fruit ripening. Excessive ethylene under stress leads to defoliation, stunted growth, and accelerated senescence.	Irfan et al., 2026
5	Gibberellins (GAs)	Drought, salinity	Breaks seed dormancy and promotes seed germination, induces shoot elongation, and initiates flowering and fruit development.	Giri and Virk, 2025
6	Brassinosteroids (BRs)	High temperature, chilling, salinity, light, drought, flooding, metals, oxidative stress, and organic pollutants.	Regulates the cellular ROS level, enhances water and nutrient absorption, membrane integrity, and osmolyte production under stress.	Ali et al., 2025
7	Jasmonates (JAs)	salinity, drought, heavy metal stress and UV irradiation'	Activates antioxidant machinery, induced systemic resistance, and molecular defense response, secondary metabolites production, regulate microbe-associated symbiotic relationships	Thakur and Yadav, 2026
8	Salicylic acid (SA)	drought, salinity, chilling, and heat.	regulation of systemic acquired resistance in plants, regulation of pathogenesis-associated protein expression, antioxidant activity.	Kostiw and Staniak, 2026
9	Strigolactones (SL)	salinity, drought, nutrient starvation, temperature, and pathogenic assail.	Regulates root colonization of microbes, enhances symbiotic nitrogen fixation, activates antioxidant defense.	Upadhyay, 2026
10	Polyamines	Low and high temperature stress, Water Stress, Salt Stress, Oxidative Stress.	Involved in various physiological processes in plants including embryogenesis, organogenesis, reproductive development, leaf senescence, and fruit maturity, stabilizes membrane, regulates ROS, and cellular signaling	Gantait et al., 2026

The modern trajectory in microbial ecology, genomics, and systems biology has expanded our understanding of plant-microbe interactions and established a new paradigm for microbiome-based agricultural innovations. To overcome the historical inconsistency of field-scale applications, there is an increasing integration of Industry 4.0 technologies. The integration of modern digital technologies, including artificial intelligence-assisted bioformulation development and Internet of Things-based monitoring systems, is further enhancing the precision and effectiveness of microbiome management approaches. These technological advances facilitate the refinement of microbial consortia, improve the stability and performance of microbial inoculants, and enable real-time monitoring of plant-microbe interactions under field conditions.

Smart-MIP (Microbial -and sensor based integrated precision) frameworks integrated microbial technologies with didigital agriculture to advance rhizosphere management. By combining beneficial inoculants, real-time environmental sensing, and predictive analysitics, Smart-MIP shifts agriculture from conventional inputs to adaptive, data-driven interventions. This unified platform improves the consistency and scalability of plant growth -promoting rhizobacteria under stress conditions (Rezaee Danesh, 2025).

In this review, we map the current frontiers of the role of PGPR in enhancing crop resilience and promoting sustainable agricultural systems under changing climatic conditions. By integrating the molecular underpinnings of transgenerational stress memory and root architecture modulation with biochemical indicators of soil health restoration, wes provide a holistic view of the plant-microbe-soil interface. Furthermore, this synthesis addresses these critical translational bottlenecks of field application by proposing a framework that leverages Industry 4.0 technologies, such as AI-assisted bioformulation and IoT-based monitoring. Ultimately, this article highlights the utility of PGPR-centered microbiome engineering as a transformative, precision-driven approach for achieving resilient crop production in the face of escalating global environmental change.

## 2 Plant Growth-Promoting Rhizobacteria (PGPR): an overview

**Figure 1:**
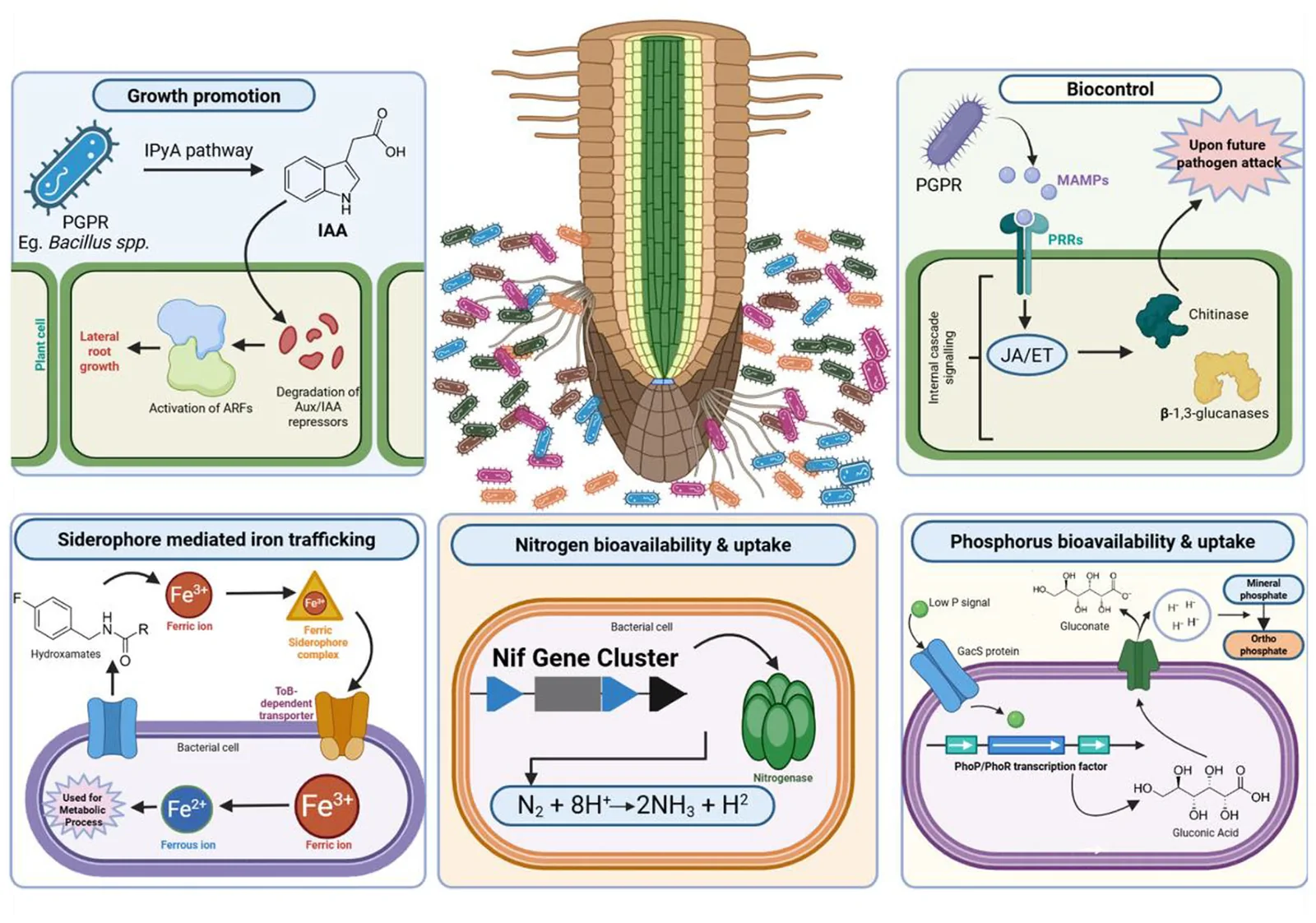
Direct and indirect mechanisms of PGPR in rhizosphere.

PGPR are beneficial bacteria that inhabit the rhizosphere, a dynamic interface where complex chemical cross-talk occurs between plant roots and microbes. This narrow soil zone is influenced by root exudates, including sugars, amino acids, and organic acids, which modulate microbial activity to improve plant health and nutrient cycling (Goswami and Deka, 2020). The efficacy of PGPR in enhancing ecosystem robustness and nutrient bioavailability is governed by complex molecular signaling and genetic regulation. PGPR directly enhance vegetal development by modulating hormonal homeostasis and orchestrating complex chemical crosstalk within the rhizosphere (Ehinmitan et al., 2024). A primary molecular mechanism involves the microbial synthesis of Indole-3-Acetic Acid (IAA) via the indole-3-pyruvate (IPyA) pathway, which triggers the degradation of host Aux/IAA repressor proteins, thereby activating Auxin Response Factors (ARFs) to stimulate lateral root proliferation (Ryu and Patten, 2008; Prasad et al., 2024). Simultaneously, these microbes bolster stress resistance through ACC deaminase activity, an enzymatic process that cleaves 1-aminocyclopropane-1-carboxylate, the immediate precursor of ethylene, to suppress stress ethylene levels and delay premature senescence (Danish et al., 2020; Shahid et al., 2023). Furthermore, diazotrophic species like *Azospirillum* facilitate biological nitrogen fixation by converting atmospheric N_2_ into assimilable NH_3_ through the nitrogenase enzyme complex, a high-energy process strictly regulated by the nif gene cluster and coordinated by the NifA/NtrC transcriptional system in response to nitrogen availability (Zhang et al., 2006). Beyond nitrogen, PGPR improve nutrient uptake potency through sophisticated chemical mobilization strategies. They solubilize inorganic phosphorus by secreting low molecular weight organic acids, such as gluconic and citric acids produced via the direct oxidation pathway, which chelate phosphate-bound cations (Oteino et al., 2015). To overcome iron limitation, these bacteria synthesize high-affinity siderophores that form ferric complexes recognized by specialized TonB-dependent transporters on the bacterial outer membrane, ensuring efficient nutrient acquisition and a competitive ecological advantage. PGPR catalyze root architecture modifications and bolster plant immunity through a sophisticated integration of direct physiological stimulation and indirect molecular defense cascades. By synthesizing essential phytohormones, including auxins, gibberellins, and cytokinins, these microbes propel root tip elongation and significantly increase the total surface area available for nutrient absorption (Shahid et al., 2023). These direct enhancements are complemented by the deployment of siderophores, high-affinity ligands (<1,000 Da) such as catecholates or hydroxamates, which sequester ferric iron (Fe^3+^) and transport it via TonB-dependent outer membrane transporters (Timofeeva et al., 2022). This creates a localized zone of iron depletion that effectively starves soil-borne pathogens, providing the PGPR and host with a competitive ecological advantage (Timofeeva et al., 2022). Furthermore, PGPR fortify the host via Induced Systemic Resistance (ISR), a process primarily regulated by jasmonic acid (JA) and ethylene (ET) signaling pathways rather than salicylic acid (Serteyn et al., 2020). This systemic defense is triggered when microbial elicitors, such as lipopolysaccharides (LPS) or N-acyl homoserine lactones (AHLs), are recognized by plant receptors, priming the host to rapidly accumulate hydrolytic enzymes like chitinases and \beta-1,3-glucanases. These enzymes actively degrade the cell walls of invading biological threats, ensuring a robust and energy-efficient defense response across the entire plant (Subhash et al., 2022). The conceptual pathways of microbe-mediated carbon and nitrogen fluxes are depicted in Figure 1.

They further expand their biocontrol repertoire through the localized secretion of potent antimicrobial metabolites and the production of specialized lytic enzymes, which directly compromise the structural integrity of competing pathogens. The molecular mechanism of antibiotic production involves the synthesis of diverse compounds such as 2,4-diacetylphloroglucinol (DAPG), phenazines, and pyrrolnitrin, often regulated by complex quorum sensing (QS) systems and the GacS/GacA two-component system (Ehinmitan et al., 2024). These antibiotics function as potent inhibitors of cellular respiration or protein synthesis in target fungi and bacteria, effectively reducing the pathogen load within the rhizosphere. In tandem, PGPR secretes a suite of lytic enzymes, primarily chitinases, β-1,3-glucanases, and proteases, which catalyze the hydrolytic cleavage of structural polymers. Chitinases specifically target the β-1,4-glycosidic bonds in the chitin-rich cell walls of phytopathogenic fungi, leading to hyphal lysis and leakage of cytoplasmic contents, while proteases degrade essential surface proteins and virulence factors (Serteyn et al., 2020). This enzymatic degradation not only neutralizes active threats but also generates carbon-rich oligomers that can be utilized by the PGPR, reinforcing their dominance in the nutrient-competitive root zone.

Beyond mere assistance, PGPR actively reconfigure the plant's physiological architecture under abiotic constraints; by modulating osmotic potential and activating sophisticated antioxidant signaling cascades, these microbes dictate the maintenance of soil-plant hydraulic integrity and prevent the collapse of metabolic homeostasis during severe desiccative stress (Hönig et al., 2018). Under the escalating pressure of the global climate crisis, soil health is increasingly compromised by nutrient depletion, salinization, and the loss of microbial diversity. Preserving this “living skin” of the Earth is critical, as healthy soils act as the primary carbon sink and the first line of defense against food insecurity (Trivedi et al., 2020). Within this framework, PGPR function as the primary functional architects of the rhizosphere, driving soil health through strategic root colonization and microbiome regulation. By acting as keystone taxa, PGPR transcend simple growth stimulation; they orchestrate the assembly of broader microbial communities, enhancing the soil's suppressive capacity against pathogens while buffering plants against climatic extremes. PGPR facilitates a state of induced systemic tolerance (IST) by catalyzing essential physiological adaptations that allow the host to maintain homeostasis under abiotic constraints (Kaushal and Pati, 2025). This process involves the strategic modulation of osmotic potential, which preserves robust soil-plant hydraulic conductivity and ensures continued water uptake during periods of moisture deficit. Simultaneously, these microbial symbionts trigger the activation of complex antioxidant signaling cascades (Meena et al., 2017). By upregulating the plant's internal defense mechanisms, PGPR effectively mitigate oxidative damage during prolonged drought, securing the structural and functional integrity of the crop in the face of erratic climatic shifts (Meena et al., 2017).

While these foundational traits, ranging from nutrient mobilization to induced systemic resistance (ISR), establish the baseline for microbial-plant symbiosis, the true utility of PGPR in the modern agriculture lies in their capacity for abiotic stress mitigation. Beyond fundamental growth promotion, these “rhizosphere architects” engage in complex physiological reconfigurations, such as Induced Systemic Tolerance (IST) and epigenetic priming, which are essential for survival of the crop under the escalating pressures of global climatic volatility (Kuzyakov and Razavi, 2019).

## 3 PGPR-mediated resilience to abiotic stress extremes

### 3.1 Enhancement of carbon sequestration potential by PGPR

Soil organic carbon (SOC) supplements overall soil health, facilitating water retention capacity and soil structure while enhancing climate-resilient traits. The net transfer of atmospheric carbon dioxide to long-lived reservoirs like vegetation, long-term biomass products, wood, and dead biomass is termed “carbon sequestration” (Mason et al., 2023). Microbial process plays a key role in the formation and stabilization of the SOC through the transformation of plant derived carbon into microbial derived residues which involves the conversion of necromass and fixation and stabilization by microbial processes. Central to this is the Microbial Carbon Pump (MCP), which describes the transformation the relatively liable carbon into microbial biomass, extracellular metabolites, and necromass that contributes to persistent soil carbon pools (Zhu et al., 2020; Mason et al., 2023). Mechanistically, the MCP promotes the “entombing effect,” where microbial metabolism converts readily degradable plant substrates to chemically transformed microbial products that are subsequently protected within the soil matrix and resistant to decomposition (Zhu et al., 2020). The efficiency of the process is governed by microbial carbon use efficiency (CUE), which determines the proportion of the carbon assimilated toward microbial growth relative to the respiratory carbon loss. Consequently, higher CUE enhances microbial biomass production and elevates the quantity of microbial mass residues entering the stable SOC pool (Mason et al., 2023; Kästner et al., 2021).

Following microbial death, microbial mass is converted to necromass, which is considered to be the major precusors of stable SOC. Compared with the undecomposed plant residues, microbial necromass contains the cell wall components that are structurally resistant, which includes peptidoglycan in bacteria and chitin in fungi which can undergo post -mortem biochemical modification and becomes resistant to decomposition (Kästner et al., 2021). Stabilization of the microbial mass occurs through complementary physical and physiochemical mechanisms. Initially, the microbial residues become physically occluded with soil microaggregates thereby restricting microbial and enzymatic access and promotes the formation of the aggregated carbon (AggC) (Mason et al., 2023; Kästner et al., 2021). Secondly, microbial metabolites and necromass fragments interact with reactive clay minerals, iron- and aluminum oxide surfaces by organo-mineral interaction, including direct ligand exchange (metal ligand coordination), cation bridging, hydrogen bonding, ionic interaction, and adsorption, which results in the formation of mineral associated organic carbon. (MAOC) which is persistent and long lived SOC fraction (Kleber et al., 2021; Kästner et al., 2021). Collectively microbial metabolic transformation efficient carbon utilization, necromass formation, aggregate occusion and organo-mineral association underpin the long term stabilization and sequestration of carbon in soils. Therefore by fostering aggregated carbon (AggC) through microaggregation and mineral-associated organic carbon (MAOC) formation, microbes with high carbon use efficiency (CUE) contribute to improved carbon sequestration (Tao et al., 2018).

**Figure 2:**
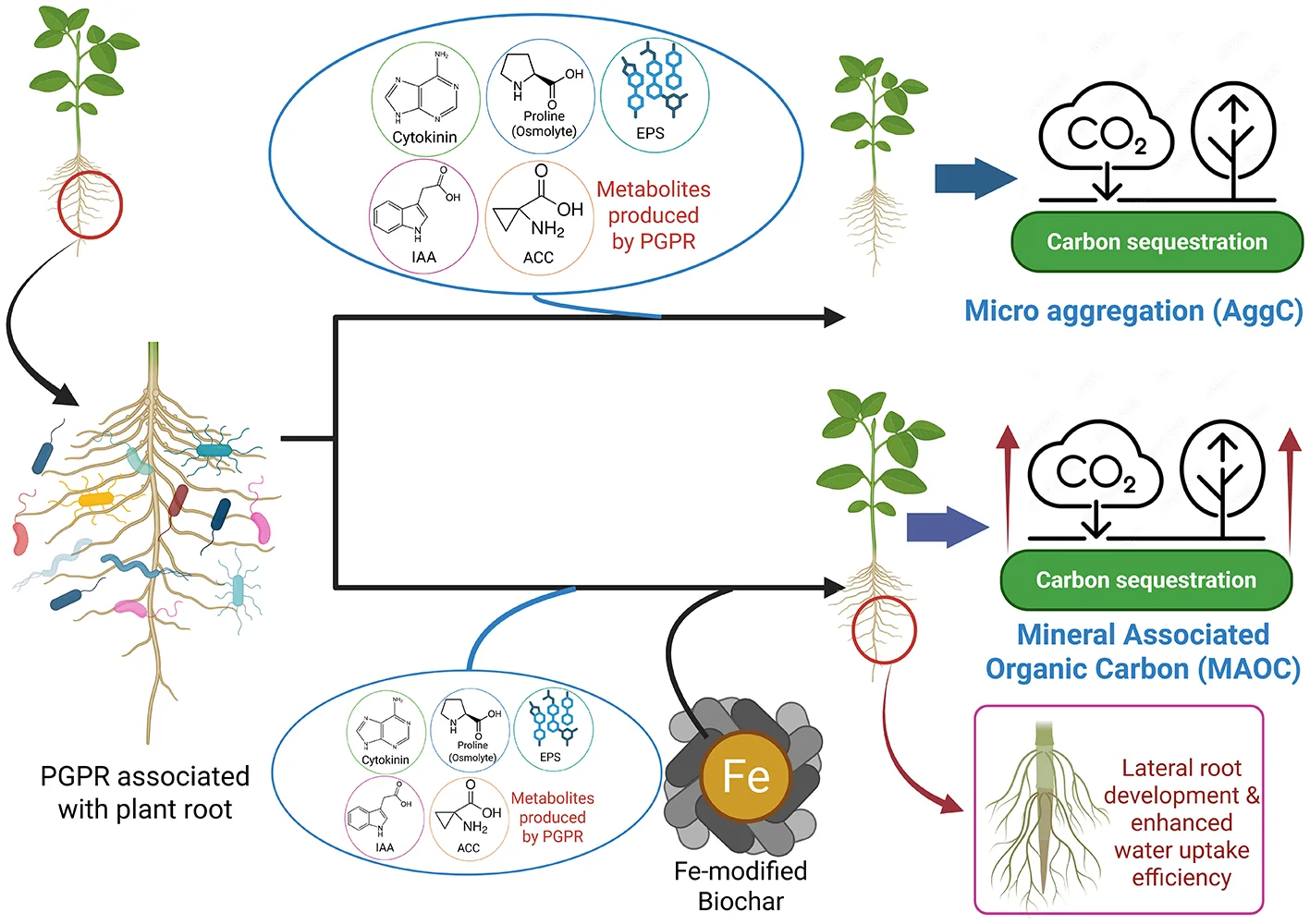
Schematic representation of PGPR mediated carbon sequestration through improved plant biomass, microaggregation,mineral associated organic carbon (MAOC), along with Fe-modified biochar, which enhances lateral root development and water uptake efficiency.

PGPR helps in the process of carbon sequestration by enhancing the secretion of plant hormones, thereby improving the root architecture and branching, stimulating root exudation, and facilitating carbon input (P. Yang et al., 2024). For instance, shoot growth and subterranean traits of plants such as rice were enhanced by inoculation of single and consortium strains such as *Bacillus subtilis* DD-4, *Bacillus megaterium* DD2, and *Bacillus aryabattai*, as they synthesize IAA, gibberellins, and phosphorus, which help survival under climate stress (Kelbessa et al., 2023; Liu et al., 2022). Building upon these, soil amendment serves as the convincing solution for PGPR-mediated carbon sequestration. The integration of PGPR with sewage sludge-based soil amendments helps in sequestering carbon. Carbon accumulation was predominantly witnessed in *Populus nigra* when inoculated with four PGPR strains, *Bacillus sp*. SK2.3, *Streptomyces sp*. SK20.12, *Massilia niastensis*, and *Rhodococcus sp*. SK12.6 under compost-amended soil (Romero-Estonllo et al., 2023). Advancement with the soil amendment strategy, biochar application also contributes to carbon sequestration. Under hypoxic conditions, carbon-rich biomass is converted to biochar, where Fe-modified biochar predominantly recruits PGPR to improve carbon sequestration (Figure 2). Biochar application fosters a conducive micro-environment that recruits keystone carbon-cycling taxa, including members of the phyla *Proteobacteria, Firmicutes*, and *Gemmatimonadota* by providing a binding site and converts CO_2_ into carbonate ions by producing carbonic anhydrase and invertase, increasing soil inorganic carbon (SIC), SOC, and particulate organic carbon (POC) (Niu et al., 2024). Additionally, bimetallic composites such as rice straw biochar with iron-manganese oxide (RSB-Fe/Mn) enhanced SOC by 16.3% in dry fields and 33.9% in flooded fields, and rice straw biochar with iron and magnesium oxide (RSB-Fe/Mg) increased it by 6.7% in dry fields and 27.2% in flooded fields of paddy. Iron oxides facilitate long-term sequestration by stabilizing soil organic carbon, enhancing MAOC,and shielding it from microbial degradation (Mabagala et al., 2025). Similarly, iron-loaded biochar, when combined with phosphorus and field mulching, improves peanut yield, water use efficiency, and root development, lending a hand to sequester carbon in soil, serving as an efficient method in semi-arid and phosphorus-deficient regions (Luo et al., 2024). Thus, these strategies highlight the exemplified role of PGPR in sequestering carbon and the interaction of soil microbes and engineered soil amendments in mitigating long-term CO_2_ levels.

### 3.2 Mitigation of greenhouse gas emission

The effectiveness of terrestrial ecosystems as a climate buffer depends equally on the attenuation of non-CO_2_ greenhouse gases, even if stabilizing soil organic carbon is a key component of climate-smart agriculture. Nitrous oxide (N_2_O) is a particularly insidious byproduct of modern agriculture. With an atmospheric lifespan of approximately 116 years and a global warming potential 265 times that of CO_2_, N_2_O emerges as a critical area for climate change mitigation in agriculture (Oke et al., 2026). The improvement of Nutrient Use Efficiency (NUE) and the direct regulation of microbial guilds involved in methanogenesis and denitrification are the main drivers of the microbial-mediated decrease of GHG emissions (Zou et al., 2025). The “nitrogen-carbon trade-off,” in which enhanced nitrogen fertilization meant to increase carbon-sequestering biomass frequently causes a disproportionate surge in N_2_O flux, has historically impeded the mitigation of these emissions (Shcherbak et al., 2014). Current mitigation strategies rely heavily on synthetic chemical inhibitors such as N-(n-butyl) thiophosphoric triamide (NBPT), which, despite their efficacy, face challenges regarding environmental persistence, microbial community shifts, and inconsistent performance across heterogeneous soil types (Sun T. et al., 2025). The efficacy of synthetic inhibitors is also hampered by their physical and chemical limitations. NBPT, for example, is highly sensitive to environmental factors; it has a half-life of <6 months at room temperature and degrades rapidly in the presence of sulfur-containing fertilizers. Its performance is also highly soil-dependent, with peak efficacy in neutral-pH and clay-rich soils, while performance drops significantly in acidic, sandy soils prone to leach (Peters and Thiele-Bruhn, 2022).

Mitigation of greenhouse gases (GHGs) in agricultural ecosystems, particularly nitrous oxide (N_2_O) emissions, is closely related to nitrogen-transforming microorganisms and their functional genes. Key microbial biomarkers such as amoA, nirK, nirS and nosZ regulate nitrification and denitrification processes. The amoA gene encodes ammonia monooxygenase, which is involved in the first step of nitrification by ammonia-oxidizing bacteria (AOB) and archaea (AOA) and regulates nitrate availability and subsequent N_2_O production (Hui et al., 2024). The nirK and nirS genes encode nitrite reductases involved in N_2_O formation during denitrification, whereas nosZ encodes nitrous oxide reductase that reduces N_2_O to harmless N_2_, and is a key biological mechanism of N_2_O mitigation (Intrator et al., 2024). Agronomic practices such as organic amendments, balanced fertilization and crop diversification can enhance nosZ containing microbial populations and nitrogen use efficiency. Furthermore, nitrogen transforming microbial networks composed of nitrifiers, denitrifiers and other rhizosphere microorganisms control ecosystem stability and nutrient cycling. The combination of functional gene analysis and microbial network approaches offers a promising approach for the development of climate-smart agricultural methods to reduce GHG emissions and improve soil sustainability (Beattie et al., 2025; Purohit et al., 2024).

**Figure 3:**
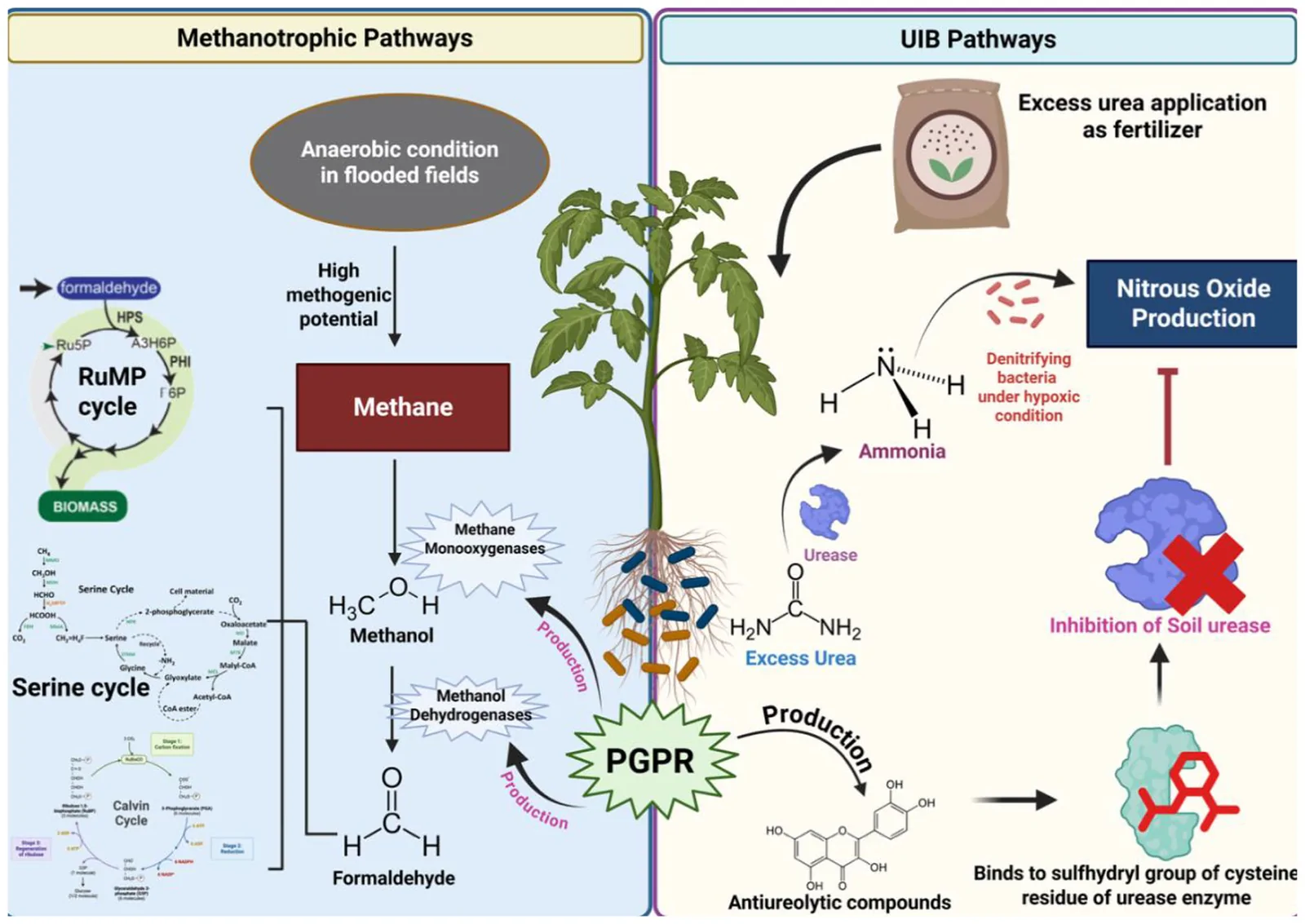
Role of PGPR in mitigating greenhouse gas emissions through methanotrophic and urease inhibition pathways.

By separating crop yield from excessive chemical inputs through the systemic management of microbial nitrogen and carbon metabolic pathways, PGPR provides a biological answer to this contradiction. PGPR reduces the pool of excess nitrates, which are the main substrates for denitrifying bacteria that produce N_2_O, by optimizing the nitrogen cycle through biological nitrogen fixation and the secretion of biological nitrification inhibitors (BNIs) (Backer et al., 2018). Concurrently, specialized PGPR consortia function as competitive inhibitors or methane-oxidizing catalysts in anaerobic conditions like flooded paddy fields, greatly reducing the rhizosphere's methanogenic potential (Bodelier and Steenbergh, 2014). This multi-gas mitigation approach highlights PGPR's function as crucial regulators of the atmosphere-biosphere interaction in addition to their role as growth promoters (Figure 3).

The escalation of global agriculture has conventionally relied on the massive application of synthetic nitrogen fertilizers, with urea representing over 55% of the total 107 million tons of nitrogen consumed annually (Oke et al., 2026). Although essential for sustaining the caloric needs of a growing population, the efficiency of urea application is staggeringly low, with losses frequently exceeding 50% due to rapid enzymatic hydrolysis. Multiple strategies have been proposed to reduce agricultural N_2_O emissions, including management of water regime, crop residues and chemical materials application. Several studies have also suggested that bioorganic fertilizers containing PGPR can reduce N_2_O emissions from agricultural soils. Application of *T. guizhouense*-based bioorganic fertilizer suppressed ammonia-oxidizing bacteria abundance and thereby downregulated nitrification and subsequent N_2_O production (Geng et al., 2021). This inefficiency is driven by soil urease, a ubiquitous nickel-dependent enzyme that catalyzes the conversion of urea into volatile ammonia. Urease serves as the primary gateway for nitrogen entry into the biosphere from urea-based sources. It is a complex multi-subunit metalloenzyme, characterized by a bis-nickel active site where two Ni^2+^ ions are bridged by a hydroxide ion and a carbamylated lysine residue (Siedt et al., 2026). The enzyme's primary function is the hydrolytic decomposition of urea into one molecule of carbamate and one molecule of ammonia. Carbamate subsequently undergoes spontaneous hydrolysis to yield a second molecule of ammonia and carbon dioxide (Meng et al., 2023).

The deployment of urease-inhibiting bacteria (UIB) for smart agriculture will act in parallel as PGPR. This bio-intervention attempts to unify nitrogen stabilization with systemic plant health improvements, offering a sustainable alternative to chemical additives (Mudzielwana, 2025). Translating the theoretical potential of urease-inhibiting bacteria (UIB) into commercial-scale agricultural tools requires overcoming significant biological bottlenecks, particularly in strain isolation, formulation, and field colonization. Identifying an ideal strain involves screening for microbes that not only exhibit targeted biochemical traits but also tolerate drastic physicochemical changes and demonstrate compatibility with native rhizobacterial communities (Basu et al., 2021). Even when highly effective strains are isolated, maintaining their viability and shelf-life presents a major formulation challenge; for instance, gram-negative rhizobacteria like *Pseudomonas* possess excellent biocontrol potential but lack protective spores, rendering them highly sensitive to desiccation and requiring advanced encapsulation technologies, such as alginate beads or nano-encapsulation, for survival and controlled release (Basu et al., 2021; Bouremani et al., 2023). Furthermore, once applied, these strains face fierce competition in the nutrient-dense rhizosphere. To establish themselves, introduced UIBs must possess high competitive saprophytic ability to outcompete native microbes for carbon-rich root exudates while simultaneously evading the plant's own immune receptors such as FLS2, which detects bacterial flagellin to avoid triggering an energy-consuming defensive response instead of a productive symbiotic relationship (Santoyo et al., 2021).

Microbial urease inhibition is mediated through multiple biochemical pathways, frequently involving the production of secondary metabolites that directly interfere with the structure and activity of the urease enzyme. PGPR, particularly members of the *Streptomyces* and *Bacillus* genera, synthesize phenolic compounds and flavonoids (e.g., quercetin, rutin, kaempferol) that exhibit strong anti-ureolytic activity by binding to sulfhydryl groups of cysteine residues proximal to the enzyme's active site or by chelating the nickel ions essential for catalytic function, thereby leading to enzyme inactivation (Sun G. et al., 2025). Additionally, certain bacteria produce hydroxamate compounds, such as acetohydroxamic acid (AHA), which act as potent inhibitors by forming stable complexes with the nickel center of urease, effectively blocking urea access to the active site. Beyond direct biochemical inhibition, urease-inhibiting PGPR (UIB-PGPR) also suppress ureolytic activity through ecological mechanisms, including competitive exclusion and niche occupation within the rhizosphere, where high population densities limit the proliferation of indigenous, high-urease-producing microorganism (Sheo and Upadhyay, 2012). Furthermore, some PGPR contribute to pH regulation by secreting organic acids or producing exopolysaccharides (EPS), thereby buffering the rhizosphere environment, mitigating rapid pH increases associated with urea hydrolysis, and promoting the stabilization of ${\text{NH}}_{4}^{+}$ over the volatilization of NH_3_ (Meng et al., 2023).

Meta-analyses of field trials spanning 2010–2024 provide robust evidence for the efficacy of urease inhibitors in reducing agricultural greenhouse gas footprints. When properly implemented, these strategies achieve an average N_2_O reduction of 42% (95% CI: 35%−50%) compared to conventional urea. The peak performance of these inhibitors is observed in clay and clay-loam soils (up to 48% reduction) and humid climates, where high cation exchange capacity (CEC) helps stabilize the inhibitors and prolong their activity (Oke et al., 2026).

Beyond urease inhibition, specialized PGPR consortia manipulate the broader denitrification and methanogenesis pathways. For example, bio-fertilizer inoculation with *Bacillus subtilis* HJ5 and DF14 increased the abundance of nirS and nosZ genes associated with denitrification, promoting efficient conversion of nitrogen intermediates and resulting in decreased N_2_O production (Tao et al., 2018). Two strains of *Stutzerimonas stutzeri* NRCB010 and NRCB025, demonstrated significant plant growth-promoting potential on agar plates, likely due to their ability to produce auxins and siderophores and to solubilize phosphate. In addition, these strains exhibited efficient nitrate-nitrogen (${\text{NO}}_{3}^{-}$-N) removal from agricultural wastewater via denitrification (Zhang et al., 2022). Concurrently, methanotrophs provide a biological pathway to mitigate methane emissions, a greenhouse gas responsible for about 30% of recent global warming. These bacteria naturally oxidize methane in aerobic environments, offering potential for engineering applications in agriculture, waste management, and atmospheric removal. With methane levels rising rapidly, leveraging methanotrophs aligns with sustainable climate strategies, bypassing high-energy chemical methods (Moreno et al., 2018). They are aerobic bacteria specialized in methane (CH_4_) oxidation, employ a highly efficient enzymatic mechanism to convert this potent greenhouse gas into less harmful compounds, preventing its atmospheric release (Saunois et al., 2020). The process begins with the initial activation of methane by methane monooxygenases (MMOs), copper- or iron-containing enzymes that catalyze the insertion of oxygen from molecular O_2_ into the stable C–H bonds of CH_4_, forming methanol (CH_3_OH). Two methane monooxygenase (MMO) variants exist: particulate MMO (pMMO), a membrane-bound, high-affinity enzyme predominant in most methanotrophs under high-CH_4_ conditions (e.g., >1% v/v), and soluble MMO (sMMO), a cytosolic diiron enzyme active at trace methane levels (<1 ppm) with broader substrate specificity but lower affinity (Guerrero-Cruz et al., 2021). Methanol is then oxidized to formaldehyde (HCHO) by methanol dehydrogenases (MDH), typically pyrroloquinoline quinone (PQQ)-dependent in Type I methanotrophs or calcium-dependent in Type II. Formaldehyde, a toxic intermediate, undergoes rapid further oxidation to formate (HCOO^−^) via formaldehyde dehydrogenase and finally to CO_2_ by formate dehydrogenase, completing mineralization. This generates reducing equivalents (NADH/FADH_2_) funneled into the electron transport chain, establishing a proton motive force for ATP synthesis (Keltjens et al., 2014). Carbon assimilation diverges by types. Type I (*Gammaproteobacteria*, e.g., *Methylomonas*) use the ribulose monophosphate (RuMP) cycle, where formaldehyde condenses with ribulose 5-phosphate to ribulose monophosphate, regenerating ribulose via transketolase and isomerase reactions (van Teeseling et al., 2014). Type II (*Alphaproteobacteria*, e.g., *Methylosinus*) employ the serine cycle, fixing formaldehyde onto serine via hydroxypyruvate and glyoxylate pathways, forming C_4_ acids like malate for gluconeogenesis. *Gammaproteobacteria* such as *Methylococcus capsulatus* combine RuMP with partial Calvin cycle elements via low levels of ribulose-1,5-bisphosphate carboxylase. Excess carbon mineralizes to CO_2_, achieving 50%−90% oxidation efficiency under optimal pH (6–8), O_2_, and copper availability, which regulates pMMO expression (Lee et al., 2004). This pathway not only mitigates CH_4_ but supports biomass growth, enabling biotechnological scaling. Thus, methanotrophic pathways transform climate threats into microbial biomass, promising scalable CH_4_ mitigation tools.

## 4 PGPR-mediated remediation of polluted soils under climate change

A complex mixture of heavy metals, microplastics, and persistent organic pollutants (POPs) have been introduced into agricultural soils by anthropogenic activities ranging from extensive industrialization to incorrect wastewater discharge (Salim et al., 2022). These pollutants do more than only directly harm plants; they also drastically change the physicochemical structure of the soil, reducing microbial diversity and upsetting vital biogeochemical cycles (Zhu et al., 2023). Variations in soil moisture and pH can increase the bioavailability and mobility of these toxins under the aggravating effects of climate change, resulting in high-stress conditions that reduce crop productivity, especially in arid and saline locations. PGPR have become sophisticated biological agent for comprehensive ecosystem restoration in this environment. Instead of functioning as separate degraders, PGPR use plant-microbe interactions to combine detoxification with nutrient cycling and soil structural repair. In contrast to traditional physicochemical treatment, which is frequently energy-intensive and environmentally harmful, this dual-functionality provides a sustainable option (Aasfar et al., 2021).

### 4.1 Heavy metal tolerance of plants with the synergy of PGPR

While these systemic disruptions undermine the soil's fundamental integrity, the specific impact of heavy metals represents one of the most persistent threats to the physical development of the crops themselves. Unlike organic pollutants, metals such as cadmium (Cd), lead (Pb), arsenic (As), and chromium (Cr) are non-biodegradable, accumulating in the soil matrix and exerting prolonged toxicity. These ions arrest cell division within the meristematic zones, leading to stunted root systems that are physically incapable of sufficient water and nutrient acquisition (Rashid et al., 2023).

Beyond the rhizosphere, HMs induce systemic physiological collapse. Chlorosis and necrosis of the foliage significantly reduce biomass, primarily because HMs compromise the structural integrity of the photosynthetic apparatus (Mohamed et al., 2025). Specifically, toxic ions like Cd^2+^ can competitively displace the central magnesium (Mg^2+^) ion within chlorophyll molecules, disrupting pigment stability and halting the electron transport (Hu et al., 2023). This biochemical interference is compounded by the overproduction of reactive oxygen species (ROS), such as superoxide radicals and hydrogen peroxide. These unstable molecules trigger lipid peroxidation of the cell membranes and induce DNA strand breaks, ultimately leading to programmed cell death (Mansoor et al., 2023). Furthermore, HMs displace essential cofactors (like Zn or Fe) in enzymes, inactivating vital metabolic pathways involved in nitrogen assimilation and respiration (Qin et al., 2024).

**Figure 4:**
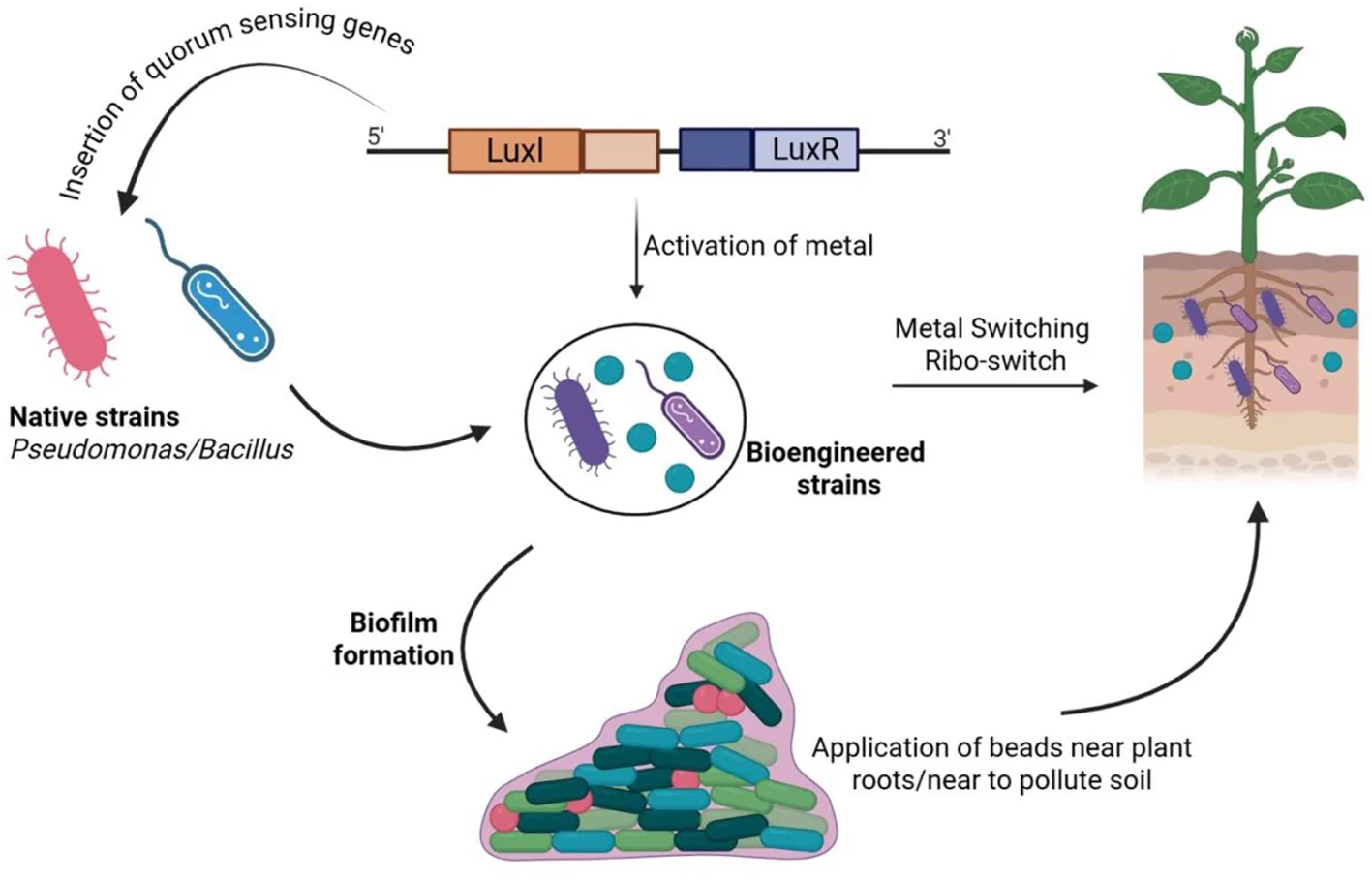
Bioengineered bacteria for soil remediation: native strains (*Pseudomonas/Bacillus*) are modified with quorum-sensing genes (LuxI/LuxR) to trigger biofilm formation.

To mitigate these threats, PGPR employ a suite of sequestration strategies, including bioaccumulation, biosorption, and biotransformation, which effectively lower the bioavailability of toxic ions (Riseh et al., 2023). Due to advances in molecular biology and genetic engineering, the effects of microbial strains have been further improved, which makes bioremediation a good substitute for traditional techniques (Riseh et al., 2023). However, traditional PGPR-based techniques often rely on constitutive gene expression, which leads to metabolic exhaustion and unintended microbial activity in uncontaminated soil zones (Abatenh et al., 2017). A paradigm shift is currently underway toward the engineering of metal-resistant PGPR equipped with quorum-sensing (QS) modules and metal-responsive riboswitches (Figure 4; Majhi et al., 2024). While this engineering strategy is promising, translating these constructs from lab to field is currently constrained by regulatory frameworks governing the environmental release of genetically modified organisms, including biosafety risk assessment and containment requirements to limit horizontal gene transfer mandatory environmental risk assessment (ERA) under instruments such as the Cartagena Protocol on Biosafety and national GMO laws, and post-release monitoring obligations (Eckerstorfer et al., 2025; Lerner et al., 2026). These genetic “on-off switches” ensure that detoxification genes are activated only when pollutant concentrations exceed a specific threshold, optimizing microbial energy expenditure. Once activated, these engineered microbes form robust biofilms enriched with extracellular polymeric substances (EPS). These EPS matrices are modified with carboxyl and hydroxyl groups, which serve as high-affinity binding sites to sequester heavy metals while simultaneously anchoring soil particles and expanding the total capture surface area (Sarkar and Bhattacharjee, 2025; Thai et al., 2023).

### 4.2 Microplastic degradation by PGPR to mitigate abiotic stress in plants

While these advanced biotechnological interventions offer a precise solution for metal toxicity, the agricultural landscape faces a simultaneous and equally pervasive threat from microplastics (MPs). MPs that are smaller than 5 millimeters are everywhere in the farming environment. They get into the soil in a few ways like when we use wastewater to water plants or when we add sewage sludge to the soil or when we use plastic to cover the ground (Souza Machado de Kloas et al., 2018). Microplastics impose a tripartite stress matrix on agricultural systems, compromising crop health through physical obstruction of soil porosity, chemical vectoring of phytotoxins, and biological disruption of the essential soil-plant-microbiome continuum (Wolff Leal et al., 2025).

By altering bulk density and pore distribution, MPs significantly change the functionality of soil. A physical barrier to root penetration and gas exchange is created by this structural interference, which also destabilizes soil aggregates and reduces the soil's ability to store water (Basumatary et al., 2025). Nanoplastics have the ability to block root cell wall pores (pits) at the cellular level, which directly prevents the uptake of water and vital macronutrients like phosphorus and nitrogen (Sarfraz et al., 2025). MPs cause systemic damage similar to heavy metal stress after they are internalized or adsorbed onto root surfaces. They do this by increasing the formation of Reactive Oxygen Species (ROS), which impair cell wall integrity and cause DNA breakage, hence drastically lowering overall plant biomass (Jia et al., 2023). MPs serve as carriers of other pollutants, which may be the most hazardous. Heavy metals, herbicides, and antibiotics from the surrounding soil are absorbed by their hydrophobic surfaces. Plants are exposed to a concentrated cocktail of poisons when they come into contact with these “charged” MPs. This exacerbates stress reactions and makes it easier for the pollutants to enter the crop's edible sections, endangering food safety (Zambrano-Pinto et al., 2024). Most importantly, MPs serve as hydrophobic carriers of additional soil pollutants. They expose plants to a concentrated “cocktail” of poisons by adsorbing heavy metals, herbicides, and antibiotics due to their high surface-area-to-volume ratio. This combination increases plant stress and makes it easier for contaminants to get into edible tissues, directly endangering food safety and human metabolic health (Wang et al., 2022).

**Figure 5:**
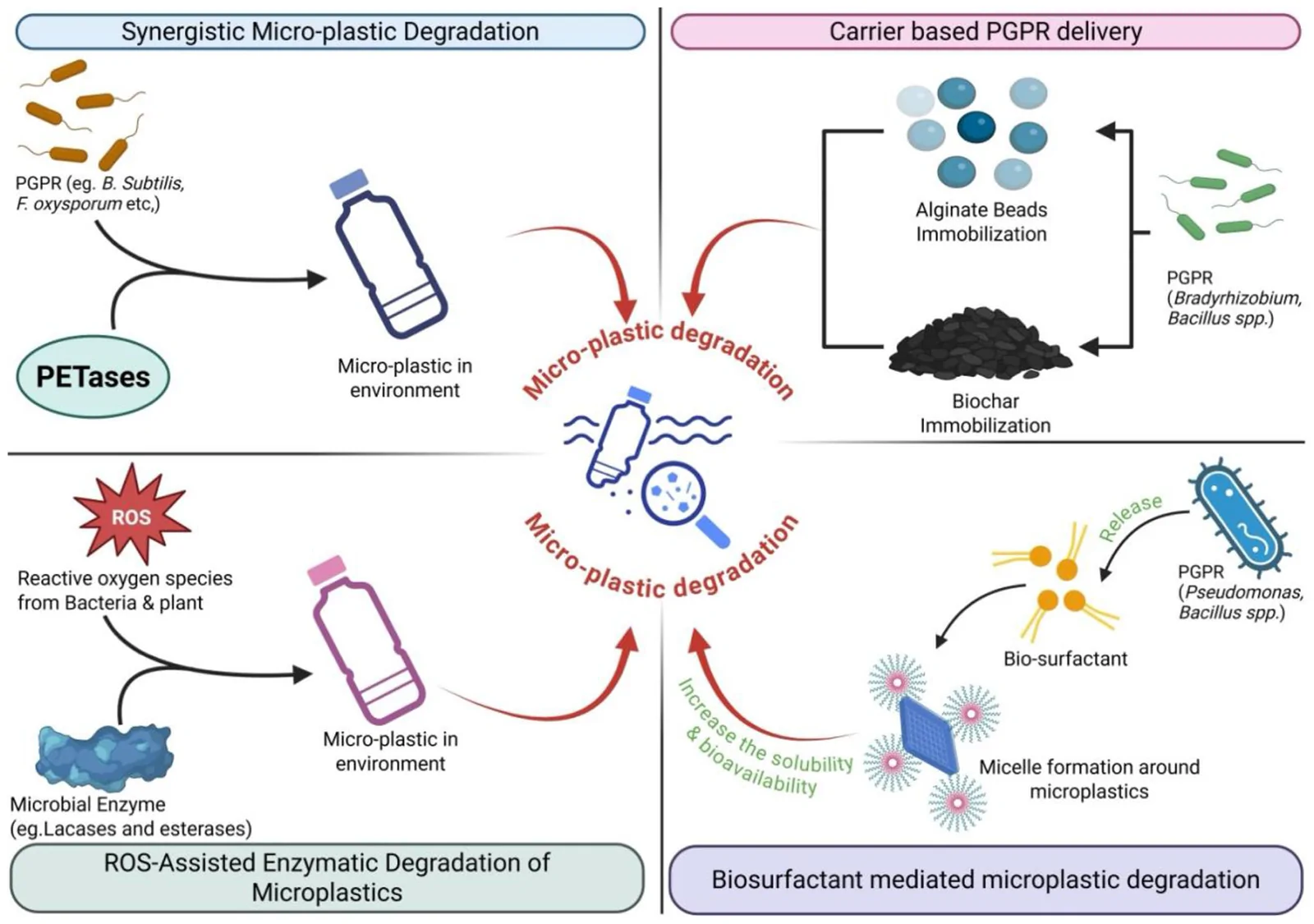
A collaborative approach for completely breaking down the microplastics occurs from the joint efforts of enzyme hydrolysis, carrier-assisted delivery, oxidative and dissolving effect of biosurfactants.

PGPR offer a promising biologically-driven pathway for MP attenuation through surface adhesion and enzymatic cleavage (Figure 5). Microbial colonization is initiated by the secretion of biosurfactants, which lower surface tension and enhance the solubility of hydrophobic polymers (Chauhan and Ambechada, 2025). Once a biofilm is established, specialized enzymes including lipases, laccases, esterases, and PETases catalyze the oxidative cleavage of polymer chains into metabolizable dimers and monomers. Recent advancements utilize PGPR-producing PETases immobilized on alginate beads to ensure controlled release and prolonged interaction with plastic residues in the rhizosphere. This enzymatic degradation is often supplemented by ROS generated during microbial metabolism, which further destabilizes polymeric chains. However, the efficacy of this “bio-cleansing” is highly dependent on environmental variables such as pH shifts and polymer crystallinity (Soursou et al., 2023; Wang et al., 2024). Despite the potential of these microbial approaches, scaling them from laboratory-controlled conditions to heterogeneous field environments remains a critical research gap, requiring further investigation into microbial stability and genetic consistency (Chandel et al., 2025; Guan et al., 2023).

Despite these promising mechanisms, PGPR based microplastic degradation still has major limitation when this is moving from lab to field. Lab studies are usually done under a clean and controlled conditions, so there will be a tendency to show better results than what actually happens outdoor. In real soil, several things get reduce how well this bacteria work, sunlight (UV) can weaken their enzymes, other soil organisms may feed on the introduced bacteria, and there are some chances where the native soil microbes compete with them for space and nutrients. Changing temperature, moisture and uneven spread of microplastics in real fields also can make results less consistent than in the lab. More specifically, introduced inoculants like *Pseudomonas putida* suffer an “establishment bottleneck” marked by quick population crashes due to the native microbial competition. Additionally, soil aggregates physically shelter microplastics from bacterial contact, and clay-humic complexes bind extracellular enzymes, deactivating them before degradation can occur. As a result of these combined biotic, physical and biochemical barriers, real-world degradation rates drop preciptously in natural field soils compared to controlled laboratory cultures. To truly confirm PGPR can work at scale, long-term- field trails under real environment conditions are needed, rather than relying only on lab based (Piekarz et al., 2026).

### 4.3 PGPR-mediated tolerance to organic contaminants

Organic Pollutants (OPs), which often coexist with plastics in agricultural soils, create a secondary tier of chemical stress that biotechnological methods for microplastic degradation must overcome. OPs include a wide range of dangerous substances that are mainly brought in by industrial runoff and wastewater irrigation, such as dioxins, persistent pesticide residues, and polycyclic aromatic hydrocarbons (PAHs). These compounds are extremely harmful, causing molecular toxicity and interfering with the basic machinery of photosynthetic processes (Desalme et al., 2013).

The immediate effects of OPs are often driven by their lipophilic nature. Once PAHs and other substances have penetrated the waxy cuticle of roots and leaves, they can build up inside membrane structures. Their internalization, disruption of the Electron Transport Chain (ETC) in chloroplasts, and inhibition of Photosystem II (PSII) efficiency led to chlorosis, poor carbon fixation, and a significant reduction in total biomass (Desalme et al., 2013). Additionally, OPs can act as “phyto-endocrine disruptors,” disrupting auxin and cytokinin hormonal signaling, which regulates seed germination and root morphogenesis. Furthermore, recent studies suggest that pharmaceutical residues in treated wastewater may alter root architecture and delay blooming, putting agricultural productivity and quality at risk (Carter et al., 2014). Advanced bioremediation approaches make use of PGPR's metabolic adaptability to reduce these systemic hazards. Numerous specialized strains have a broad range of catabolic enzymes, such as peroxidases, oxygenases, and dehydrogenases, which catalyze the oxidative breakdown of complex contaminants into simpler, non-toxic compounds.

*Pseudomonas* species in particular are well known for their ability to mineralise polycyclic aromatic compounds and encourage plant development (Loeschcke and Thies, 2015). Other notable examples include *Serratia* species that effectively mineralise the organophosphate insecticide chlorpyrifos (Wepukhulu et al., 2024) and *Bacillus* species that reduce the phytotoxicity of pyrethroid insecticides (Zhao et al., 2019). Furthermore, the catabolic activity of *Burkholderia* spp. has been shown to aid in the degradation of refractory alkanes and PAHs in contaminated environments (Revathy et al., 2015).

The degradation of OPs and Persistent Organic Pollutants (POPs) is a necessary condition for human health. Due to their propensity to bioaccumulate and bio magnify within food chains, these chemicals provide long-term risks for human immunological, neurological, and cancer illnesses. By lowering soil phytotoxicity and restoring microbial biodiversity, PGPR-mediated remediation not only restores ecosystem health but also ensures the production of safe, toxin-free agricultural commodities (Guo et al., 2021; Mateescu et al., 2024).

## 5 PGPR-driven root architecture and water efficiency under stress

While the remediation strategies discussed previously address the external detoxification of the soil matrix, the ultimate resilience of agroecosystems under climate volatility depends on the plant's internal capacity to maintain hydraulic integrity and nutrient flux. PGPR transition from “soil cleaners” to “rhizosphere architects,” actively reconfiguring the plant's physiological and structural blueprint to withstand abiotic constraints. This section explores how microbial interventions synchronize root morphogenesis with systemic defense mechanisms to mitigate the cascading effects of salinity, drought, and residual toxicity.

Under the dual pressures of salinity and heavy metal toxicity, PGPR provide a survival advantage by stabilizing the host's osmotic adjustment and ionic balance. By modulating plant ion transporters, these microbes maintain a favorable potassium-to-sodium (K^+^/Na^+^) ratio, preventing the cellular “sodium toxicity” that typically arrests growth in saline environments. Simultaneously, microbial secretions of specific chelators and osmoprotectants (such as proline and trehalose) fortify the plant's antioxidant machinery, effectively neutralizing the oxidative “burst” triggered by heavy metals (Nawaz et al., 2020; Yang et al., 2024).

Similarly, mitigation of drought stress is characterized by a sophisticated microbial-driven reconfiguration of the root-soil interface. PGPR enhances the production of key phytohormones, specifically Indole-3-Acetic Acid (IAA) and Gibberellic Acid (GA), which maximize water uptake efficiency by stimulating lateral root proliferation and elongation, thereby increasing root surface area under drought. This structural shift is further protected by ACC deaminase, a microbial enzyme that cleaves the precursor of “stress ethylene,” thereby reducing endogenous ethylene levels and alleviating ethylene -mediated inhibition of root growth under drought. (Bouremani et al., 2023). Additionally, Abscisic Acid (ABA) maintains drought-induced root morphogenesis by primary root elongation, lateral root density, and root biomass while inducing aquaporin expression (Alwutayd et al., 2023; Arkhipova et al., 2022).

The majority of PGPR influence root architecture by secreting low-molecular-weight signaling molecules. A prime example is 2,4-diacetylphloroglucinol (DAPG), produced by fluorescent Pseudomonas species, which acts as a chemical messenger to modulate root branching and density (Bouremani et al., 2023). Drought-tolerant taxa, including *Paenibacillus polymyxa* and *Azospirillum* brasilense coordinate in the synthesis of Extracellular Polysaccharides (EPS) thereby contributing to the microbial persistence and biofilm formation. This ultimately leads to bacterial adaptation during water limited condition (He et al., 2021; Ilyas et al., 2020).

These bacterial EPS secretions perform a dual role: they improve the Relative Water Content (RWC) of the plant by preventing electrolyte leakage and serve as a “biological glue” that fosters soil aggregation. This aggregation enhances the soil's water-retention capacity and porosity, ensuring that the moisture remains available to the newly “architected” root system (Bouremani et al., 2023). Although many PGPR modify the root architecture through phytohormone-mediated regulation of root elongation, lateral root development and root hair development, some strains enhance drought tolerance through physiological mechanims including stomatal closure, hydraulic maintenance, antioxidant responses and rhizosphere hydration thereby preserving root function rather than the directly altering the root structure (Grover et al., 2021; Ahmad et al., 2022; Yang et al., 2024; Bouremani et al., 2023). Through these integrated mechanisms, PGPR improves water uptake efficiency, enhance root architecture and maintain plant water status under drought conditions, as summarized in (Table 2). Beyond experimental studies, recently developed global soil -water isotope dataset provide valuable resources that help in quantifying plant water uptake and water use efficiency under diverse environmental conditions and facilitate future validation of PGPR mediated improvements in root and water acquisition and water use efficiency under drought conditions (Yang J. et al., 2026).

**Table 2:** PGPR strains and their mechanism contributing in root structure modification, relative water content and water use efficiency under drought stress.

S. No	PGPR strains	Crops	Effects	Mechanism	References
1.	*Flavobacterium sp., GJW24*	*Arabidopsis, brassica*	Improves drought resistance, enhanced ABA mediated stomatal closure, upregulation of genes associated with root architecture	ABA mediated stomatal closure limits the transpirational water loss and PGPR derived IAA and ACC deaminase promotes lateral root and root hair development whereas EPS enhances the root-soil contact and root surface area for water uptake growth	Grover et al., 2021; Kim et al., 2023; Teng et al., 2023
2.	*Enterobacter cloaca BHUAS1*	Wheat *(Triticum aestivum)*	Increases the RWC, leaf hydration, and root biomass	IAA contributes in development of lateral root and root hairs whereas EPS improves rhizosphere water retention and ACC deaminase reduces the ethylene stress and maintains root growth.	Shankar and Prasad, 2023; Grover et al., 2021
3.	*Pseudomonas mandelii IB -Ki14*	Barley (*Hordeum vulgare*)	Maintains plant water uptake under drought improves water status	Modulates the ABA homeostatis and enhances expression of HvPIP2 aquaporins leading to improved root hydrolic conductivity.	Arkhipova et al., 2022; Grover et al., 2021
4.	*Psedomonas putida KT2440*	Tomato (*Solanum lycopersicum*)	Enhanced drought tolerance through improved water use efficiency, physilogical performance and drought responsive transcriptional priming	Biofilm formation creates a hydrated biosphere,enhances root -soil contact and water retention thereby supporting root growth and function under drought while priming drought responsive transcription,and antioxidant metabolism.	Mekureyaw et al., 2026; Grover et al., 2021
5.	*Pseudomonas chloraphis O6*	*Arabidopsis*	Reduces transpiration through stomatal closure and enhances drought tolerance	Production of VOC promotes NO (nitric oxide) signaling leading to stomatal closure	Meel and Saharan, 2024; Ahmad et al., 2022
6.	*Bacillus Subtilis ALC_02*	*Arabidopsis thaliana*	Enhanced lateral root growth, root hair development and root surface area	Produces IAA and modulates auxin accumulation. Root architectural changes mediated through AUX1-, PIN2-, AXR1 dependent signaling leads to root development and lateral root growth	Jensen et al., 2024; Ahmad et al., 2022
7.	*Streptomyces pactum Act 12*	Wheat (*Triticum aestivum*)	Increased total root length, Improves root elongation and promotes deeper rooting	Regulation of auxin (AUX1-ARF7-LBD16), ABA (PP2C-SnRK2) cytokinin, GA and ethylene (ETR1-EIN2-CTR1) signaling promotes root elongation and drought adaptation. Enhanced proline accumulation, reprogramming of glycolysis, and TCA cycle promotes root development under drought	Zhang et al., 2025
8.	*Bacillus VelezensisB26*	*Brachypodium distachyon*	Increased root volume, root weight and aids in altered root architecture.	Modulates auxin and gibberelins homeostatis by altering the endogenous hormone levels by regulating auxin and gibberellin biosynthesis/gene expression thereby promoting root architecture	Sharma et al., 2022
9.	*Pseudomonas argentinensis SA190*	*Arabidopsis thaliana*	Maintains primary root growth and lateral root development under drought stress.	ABA-dependent signaling maintained root function activating ABA responsive aquaporin genes through H3K4me3 mediated transcriptional priming and improves water uptake efficiency and drought tolerance	Alwutayd et al., 2023
10.	*Bacillus subtilis* and *Azospirillum brasilense*	Wheat (*Triticum aestivum*)	Improved germination, elevates water potential, osmotic adjustment and drought tolrence	Production of EPS, IAA, GA, ABA enhances osmotic adjustment, improves rhizosphere water retention contributing to root growth and drought tolerance	Ilyas et al., 2020

Reported effects are primarily derived from cited experimental studies, while supporting review literature has been used to complement the evidence. Mechanistic explanations are integrated from both corresponding studies and established PGPR literature to explain their role in root architecture and water relations to combat drought stress.

## 6 Epigenetic modulation of plant stress responses by PGPR

Apart from the exemplified role of enhancing the physical and chemical characteristics of plants, PGPR exerts a valuable molecular effect through epigenetic regulation. When plants experience biotic or abiotic stress, they regulate their gene expression by DNA methylation, histone modification, and non-coding RNA pathways, mechanisms that constitute a biological “memory” for stress adaptation (Miryeganeh, 2025). While the full complexity of this microbial-host crosstalk remains an active area of discovery, PGPR acts as external epigenetic triggers that “prime” the plant for future stress.

PGPR colonizes the root and secretes an array of signaling molecules (elicitors, hormonal analogs, and exogenous enzymes) that interact with the plant's epigenetic machinery. These molecules influence the activity of DNA methyltransferases (e.g., DRM2, CMT3) and demethylases (e.g., ROS1, DML3, DME) effectively “switching” genes on or off without altering the underlying DNA sequence. A critical example is observed in *Medicago truncatula*, where the high expression of the DNA demethylase DEMETER (mtDME) is essential for root nodule formation, highlighting that epigenetic fluidity is a prerequisite for successful symbiotic relationships (Zou et al., 2025).

Additionally, Histone modification helps regulate the accessibility of chromatin and availability of genes for transcription (Zou et al., 2025). PGPR triggers low-level immune signals or is involved in induced systemic resistance and activates the expression of transcription factors like WRKY, MYB, and NPRI, which promote plant immunity (Salwan et al., 2023; Ding et al., 2022). Transcription factors interact with the histone-modifying enzyme complex, leading to epigenetic modification (Su et al., 2024). WRKY or TGA transcription factors associate with the histone acetyltransferases mediated regulatory mechanism, leading to transcriptional activity (Javed and Gao, 2023; Yun et al., 2024; Shih et al., 2025). In addition, Microbial Volatile Organic Compounds (VOCs) also serve as potent epigenetic regulators. In *Paenibacillus polymyxa* CR1 helps regulate the expression of drought resistance genes like RD29A and RD29B through histone modification and chromatin remodeling, enhancing the systemic tolerance and plant survival in drought in *Arabidopsis thaliana* (Liu et al., 2023).

PGPR further refine plant defense by regulating the biogenesis of small and long non-coding RNAs (lncRNAs). Microbial inoculation has been shown to activate the specific endogenous miRNAs miR88469, miR1172, and miR397, which regulate immune response and contribute to resistance against environmental stress (Zou et al., 2025). In cereal crops like rice, specific lncRNAs interact with NAC transcription factors; for example, SNAC3 and ONAC022 have been identified as key regulators that synergistically improve both drought and salinity tolerance. Through these sophisticated molecular interventions, PGPR do not merely provide a temporary boost to plant health; they reprogram the plant's regulatory landscape, fostering a state of enhanced “readiness” that allows the host to adapt more efficiently to the escalating pressures of climate change (Salunke et al., 2025).

## 7 Impact of PGPR on soil health and ecosystem functionality

While previous sections detailed how PGPR reprogram a plant's internal molecular machinery, the ultimate success of these interventions depends on the biochemical restoration of the surrounding soil matrix. Beyond their role in nutrient solubilization, PGPR function as biological engineers that convert loose, degraded substrates into robust, aggregated environments through a synergistic triad of enzymatic mechanisms.

### 7.1 PGPR Enzymatic Activity (EA) for enhanced soil health

**Figure 6:**
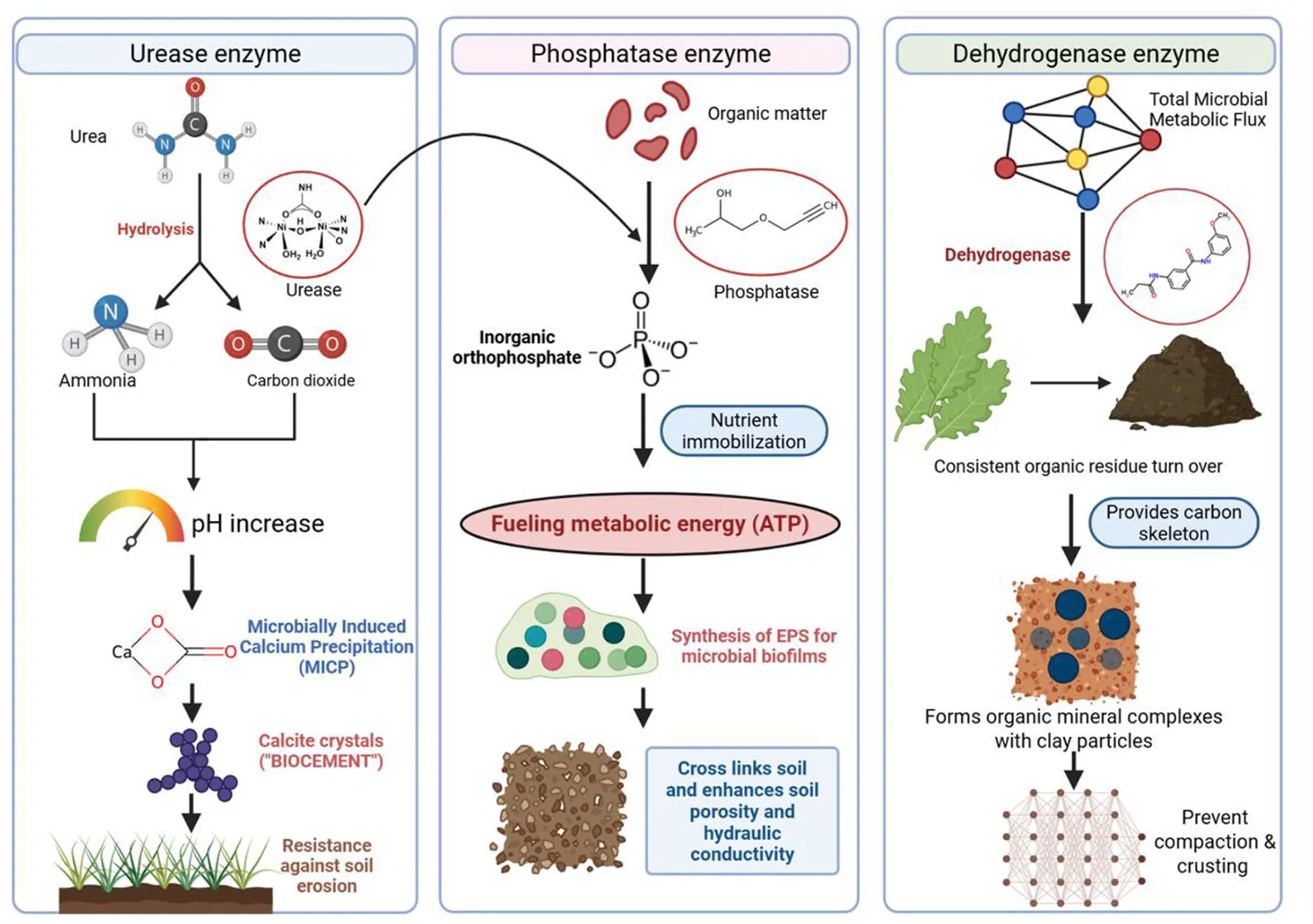
Synergistic enzymatic triad in soil structural restoration. Schematic showing the roles of **(Left)** Urease in urea hydrolysis and pH elevation to drive MICP-based bio cementation for erosion resistance; **(Center)** Phosphatase in mobilizing inorganic phosphorus for ATP and EPS synthesis, enhancing macro-aggregation and hydraulic conductivity; and **(Right)** Dehydrogenase as a metabolic proxy for organic residue turnover, providing carbon skeletons for organo-mineral complexes to prevent compaction and crusting.

The biochemical restoration of soil structural stability is facilitated by a synergistic triad of enzymatic mechanisms that convert loose substrates into robust, aggregated matrices. Urease (EC 3.5.1.5) is the main enzyme that makes this change happen, which is involved in the breakdown of urea into NH_3_ and CO_2_ (O'Donnell et al., 2023). This reaction causes the pH to rise in the vicinity, which helps Microbially Induced Calcium Carbonate Precipitation (MICP) to occur. These calcite crystals work like “bio cement,” holding sand and silt particles together as in stable micro-aggregates that are very resistant to water erosion and mechanical dispersion (Iqbal, 2026). Phosphatase, which breaks down inorganic orthophosphate from organic matter, works with this structural “welding” (Y. Wang et al., 2023). This nutrient mobilization is necessary to make the metabolic energy (ATP) needed to make extracellular polymeric substances (EPS) (Costa et al., 2018). These microbial biofilms act as bridge-forming agents, linking primary soil particles together to make water-stable macro-aggregates (Figure 6). This makes the soil more porous and allows water to flow through it more easily. At the same time, dehydrogenase is the most important intracellular proxy for total microbial metabolic flux (Cycoń et al., 2010). High dehydrogenase activity makes sure that organic residues are being broken down and rebuilt, which provides the carbon skeletons needed to make organo-mineral complexes with clay particles (Amoako et al., 2025). These enzymes form a self-reinforcing loop that keeps the rhizosphere's metabolic respiration going. Phosphatase and dehydrogenase facilitate metabolic respiration, while urease offers structural support. Recent research indicates that this enzymatic synergy is the principal factor responsible for the formation of long-term structural “skeletons” in degraded soils, inhibiting compaction and crusting. Ultimately, assessing PGPR solely as nutrient solubilisers neglects their significant function as biological architects that can restore the physical and biochemical equilibrium of the soil ecosystem.

However, a research gap remains on the sustained effectiveness of these inoculants. While they may temporarily boost enzyme activities (such as dehydrogenase and urease), the long-term displacement of indigenous, poorly characterized microbial taxa remains a concern. This review also critiques the prevailing “bio-fertilizer” narrative, arguing that PGPR should instead be evaluated as soil stabilizers. We examine whether high-density microbial inoculation truly regenerates degraded soil ecosystems or if the observed benefits are merely transient physiological spikes that mask a decline in native microbial diversity (Liu et al., 2026).

From the standpoint of microbial invasion ecology, for instance, the introduction of aggressive colonizers, like certain strains of *Bacillus* or *Pseudomonas* at artificially high densities, can monopolize carbon exudates derived from plants, thereby competitively excluding slower-growing native keystone taxa (Mallon et al., 2015). Additionally, although these inoculants are meant to suppress pathogens, their secretion of bacteriocins and broad-spectrum secondary metabolites may unintentionally be toxic to native symbiotic networks, such as endemic rhizobia and arbuscular mycorrhizal fungi (AMF) (Mawarda et al., 2020). A powerful “priming effect,” in which the hyperactive inoculants speed up the mineralisation of stable soil organic carbon (SOC) to satisfy their high metabolic energy demands, can also be triggered by this enormous inflow of microbial biomass (Kuzyakov and Razavi, 2019). As a result, this metabolic explosion frequently leaves the soil environment temporarily carbon-depleted, starving local oligotrophic bacteria until the inoculant population eventually falls, even though overall enzymatic assays (such as dehydrogenase and phosphatase) may report a brief jump. In the end, the fragmentation of native co-occurrence networks and the consequent loss of long-term functional redundancy are not captured by these bulk enzymatic measurements.

The transition from traditional bio-fertilization to sustained soil ecosystem resilience hinges on the complex modulation of the soil's enzymatic landscape, moving beyond simple yield metrics to evaluate the “biological markers” of soil health. While standard reviews highlight broad benefits, a critique of recent data from 2021–2026 reveals that the efficacy of these microbes is governed by specific proxies like urease, dehydrogenase and phosphatase, which quantify the soil's metabolic “breathing.” For instance, inoculation with *Enterobacter asburiae* strain R31 has been shown to significantly elevate urease levels to 7.07 μg urea hydrolysed g^−^^1^ h^−^^1^ and dehydrogenase to 6.56 μg TPF g^−^^1^ h^−^^1^ (Baghel et al., 2025). However, a critical gap remains in understanding whether these enzymatic spikes represent a genuine restoration of the indigenous microbial community or a transient metabolic burst dominated solely by the inoculant. This “efficacy gap” is particularly evident in stress-mitigation research. *Acinetobacter radioresistens* can boost ACC-deaminase activity by up to 78.8% under high salinity (150 mM NaCl) to prevent root inhibition, the long-term metabolic cost to the plant and the potential disruption of native signaling pathways are often overlooked (Pérez-García et al., 2026). Similarly, while *Acinetobacter johnsonii* (SUA-14) reported a 3.04-fold increase in alkaline phosphatase in saline soils (Shabaan et al., 2022). Synergistic studies involving *Pseudomonas* and *B. megaterium* and soil amendments like Super Absorbent Polymers (SAP) further underscore those microbial enzymes such as sucrase and catalase, cannot function in a vacuum; they require a stable soil physical architecture to maintain the hydraulic conductivity necessary for carbon cycling (Jing et al., 2024). Ultimately, evaluating PGPR through isolated enzymatic spikes is reductive; a multi-generational, longitudinal critique is required to determine if these consortia truly regenerate degraded ecosystems or merely provide a physiological mask for declining soil biodiversity (Kaushal and Pati, 2025).

### 7.2 PGPR-mediated soil restoration in marginal lands: insights from bioenergy cropping systems

The expansion of agriculture into marginal lands under climate change has created a unique experimental framework to understand how plant-microbe interactions function under extreme constraints. Unlike conventional cropping systems, where soil fertility is externally maintained, marginal environments expose the intrinsic capacity of biological systems, particularly rhizosphere microbiomes, to sustain plant growth. In this context, bioenergy crops such as switchgrass, sorghum, and sugarcane are not merely alternative feedstocks but serve as model systems for studying PGPR-mediated soil restoration under stress conditions (Gagné-Bourque et al., 2015). Aquatic and semi-aquatic systems broaden this viewpoint. The utilization of water hyacinth in polluted settings underscores the dual benefits of PGPR-enhanced plant systems in biomass production and environmental restoration, particularly via increased pollutant tolerance and enhanced nutrient uptake (Anand et al., 2019). Microbial inoculation has been linked to better stand establishment and persistence in nutrient-poor soils in perennial grasses like switchgrass. This suggests that PGPR could help keep long-term cropping systems stable in less-than-ideal conditions (Davidson and Niepa, 2022). The PGPR-mediated enhancement of root growth and rhizosphere activity can also contribute directly to soil carbon recovery in marginal lands in addition to improving crop establishment. Higher root biomass and rhizodeposition constantly add more carbon, stimulate microbial activity, help the formation of microbial residues, and contribute to the stabilization of Soil Organic Carbon SOC. Perennial bioenergy crops like switchgrass and Silver grass have extensive root systems that contribute to carbon sequestration via plant-derived and microbial-derived carbon pools. Root-associated microbial processes increase soil aggregation and long-term carbon persistence. Together, these mechanisms improve soil structure, nutrient retention and ecosystem functioning, suggesting that PGPR-assisted bioenergy cropping systems can serve as platforms for biomass production and biological drivers of soil restoration in degraded environments (Martinez-Feria and Basso, 2020). An increasing body of evidence indicates that PGPR-associated bioenergy systems enhance soil carbon sequestration and soil functionality. However, few studies have measured the long-term effects of these interactions on SOC stability, microbial community resilience, aggregate stability and nutrient cycling under different marginal land types. Most studies look at how PGPR-mediated interactions affect plant growth or biomass yield. Fewer studies look at how they affect soil carbon dynamics, the resilience of microbial communities, or the long-term restoration of fertility in degraded environments. This gap is particularly significant for bioenergy systems, as the quality and composition of biomass, rather than merely yield, dictate its processability (Chandel et al., 2025). Recent evidence indicates that microbial associations may influence lignocellulosic characteristics under stress; however, this domain requires further investigation. Another important thing to remember is that not all marginal soils are the same. They have different physical and chemical limits and ecological histories. The effectiveness of PGPR appears to be heavily influenced by context, including plant genotype, soil type, and environmental stressors (Al-Turki et al., 2023). This variability underscores the imperative to shift from single-strain inoculation techniques to tailored microbiome management strategies, particularly for large-scale applications. When viewed as a whole, bioenergy cropping systems demonstrate that PGPR-mediated plant-microbe interactions can serve as biological engines of soil recovery. This implies that degraded land can be utilized productively with minimal chemical inputs. However, their full potential extends beyond biomass enhancement to fostering soil ecosystem resilience and sustainability. Consequently, forthcoming research should prioritize the correlation between crop-level responses and long-term soil health indicators, thereby establishing a more cohesive framework for the application of PGPR in climate-stressed agricultural systems.

## 8 Smart design and precision application of PGPR for soil health and climate change mitigation

**Figure 7:**
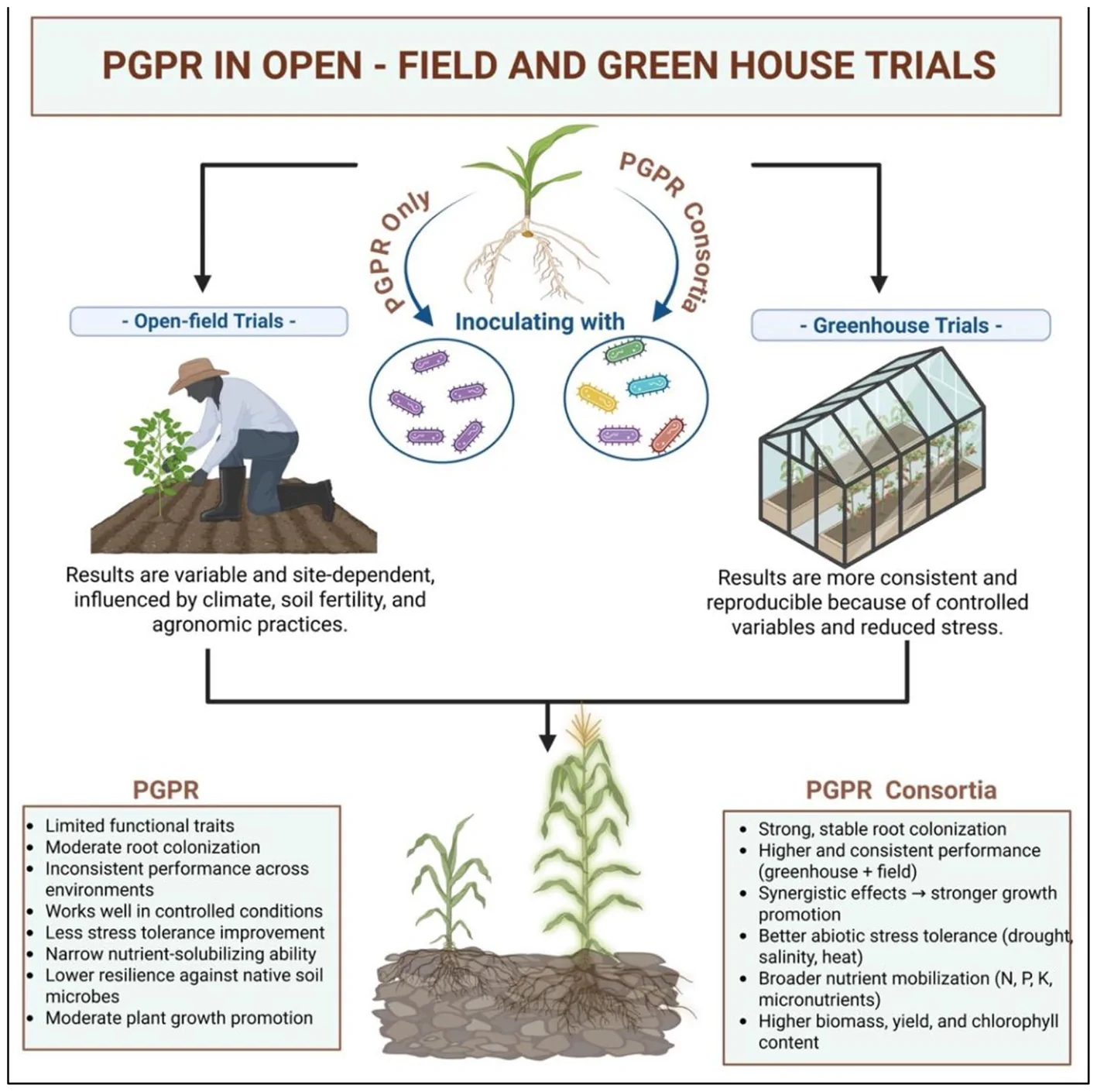
Representation of results of plant growth using PGPR and PGPR consortia in open-field vs. greenhouse trials.

The successful translation of plant growth-promoting rhizobacteria (PGPR) from laboratory research to field-scale applications remains a critical challenge in sustainable agriculture. While the functional potential of PGPR in enhancing plant growth and restoring soil health is well established, their inconsistent field performance under variable environmental conditions has limited widespread adoption (Backer et al., 2018; Calvo et al., 2014). PGPR's field application and greenhouse trials have shown encouraging but inconsistent results as it moves from the lab to the real world (Figure 7). For example, in both greenhouse and open-field settings, formulations of *Bacillus subtilis* and *Pseudomonas fluorescens* considerably decreased the incidence of wilt and increased tomato yield (Bellotti et al., 2023). Similarly, during multi-location trials, when compared to inoculation with the *Azospirillum brasilense* Sp7 strain alone, co-inoculation of *A. brasilense* Sp7 strain with promoter consortia resulted in a 2.5-fold increase in root colonization and a 27%−33% increase in maize seedling height, indicating significantly improved nitrogen uptake and biomass accumulation (Parveen et al., 2023). However, due to complex interactions with the native microbiota, erratic environmental conditions, and formulation stability, field efficacy often falls short of laboratory results. This limitation is particularly pronounced under climate change, where fluctuations in temperature, moisture, and soil chemistry directly influence microbial survival, colonization, and activity (Singh et al., 2022; Trivedi et al., 2020). Consequently, recent research has shifted toward the smart design and precision delivery of PGPR bioformulations, integrating advances in material science, microbial ecology, and digital agriculture.

### 8.1 Challenges in PGPR commercialization

A substantial number of commercially valuable PGPR strains show sharp population declines shortly after application, driven by competition for nutrients and colonization sites, predation by protozoa, bacteriophage attack, and abiotic stresses such as drought, salinity, and temperature extremes. As these populations decline, so too do the plant growth-promoting functions they were introduced to deliver: nitrogen fixation, phosphate solubilization, phytohormone synthesis, and induced systemic resistance among them, resulting in the inconsistent field outcomes that have long frustrated growers (Backer et al., 2018; Compant et al., 2019).

There is also the question of ecological compatibility; PGPR are typically viewed as environmentally benign, but that assumption deserves scrutiny: introduced strains can still reshape native microbial diversity, alter nutrient cycling, and shift ecological interactions within the rhizosphere in ways that are not immediately obvious. Ecological risk assessment therefore needs to look well beyond short-term plant growth metrics and extend to long-term monitoring of microbial community structure, functional diversity, horizontal gene transfer, and overall ecosystem stability. Fortunately, tools such as metagenomics, metatranscriptomics, and microbiome profiling now make this kind of comprehensive field-level evaluation genuinely feasible (Trivedi et al., 2020).

Beyond the biology, commercialization and regulatory hurdles present their own set of obstacles to large-scale adoption. Commercial biofertilizer efficacy tends to vary considerably depending on crop genotype, soil type, climate, agronomic practice, and formulation quality variables that are difficult to control for across different markets. Compounding this, regulatory frameworks differ widely from country to country in how they handle strain identification, biosafety evaluation, quality control, efficacy testing, and product registration, which creates real friction for companies trying to commercialize products globally. Addressing this will require standardized production protocols, robust quality assurance systems, multi-location field validation trials, and greater harmonization of regulatory frameworks, all of which would go a long way toward building farmer trust and enabling broader adoption of PGPR-based products (Malusá and Vassilev, 2014; O'Callaghan, 2016).

Looking ahead, the field would likely benefit from integrating precision agriculture approaches, synthetic microbial consortia, microbiome engineering, and AI-assisted formulation design to improve inoculant performance across diverse agroecosystems. Pairing multi-strain consortia with advanced carrier technologies and site-specific application strategies holds particular promise for improving persistence, ecological compatibility, and overall field efficacy developments that could meaningfully accelerate the commercialization of next-generation PGPR biofertilizers (Berg et al., 2020; Singh et al., 2022).

Formulation stability adds another layer of difficulty. For a bioinoculant to work at all, it has to survive manufacturing, storage, transport, and application while retaining a viable cell density high enough to establish root colonization. Traditional carriers like peat, talc, lignite, and vermiculite tend to fall short here, offering limited shelf life and poor microbial survival once storage conditions drift from ideal. This has pushed research toward more sophisticated delivery platforms, including polymer-based carriers, biochar, alginate encapsulation, hydrogels, and nanomaterial-assisted systems, all aimed at improving microbial protection, enabling controlled release, and building in greater environmental resilience (Bashan et al., 2014).

**Figure 8:**
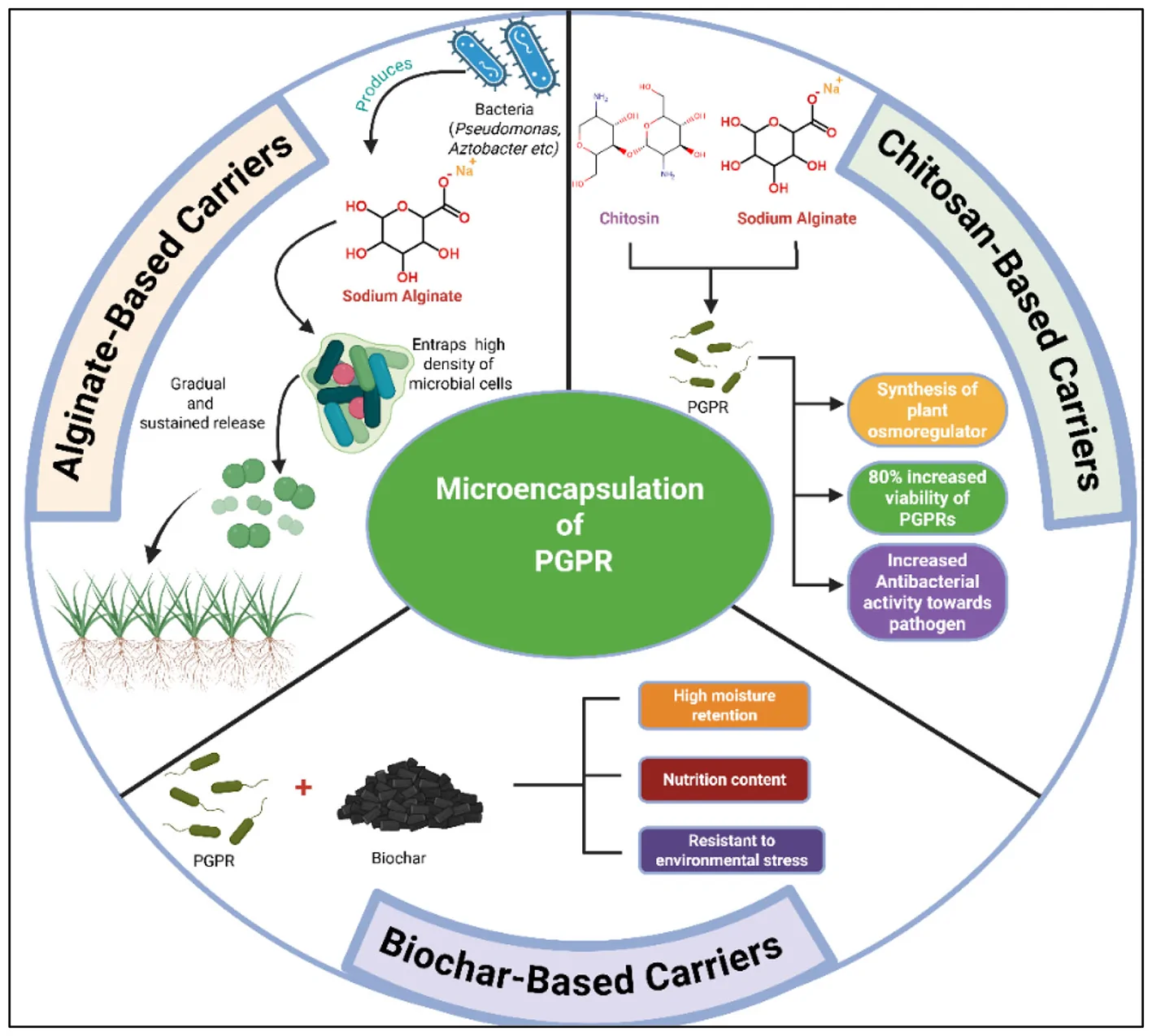
Different methods for bioformulations.

A central aspect of this transition is the development of engineered bioformulations that ensure microbial stability, targeted delivery, and sustained activity in soil systems (Figure 8). Carrier-based formulations using materials such as biochar, alginate, and lignite provide protective microenvironments that enhance microbial persistence while simultaneously contributing to soil structural improvement and carbon sequestration (Kour et al., 2020; Vassilev et al., 2020). Notably, biochar-based systems offer a dual advantage by acting as both a microbial carrier and a soil amendment, improving water retention, nutrient-holding capacity, and microbial habitat quality, key attributes for soil health restoration under climate stress (Paustian et al., 2016).

Encapsulation technologies have further advanced the field by enabling controlled release and environmental buffering of PGPR, thereby improving their functional efficiency in harsh field conditions. Approaches such as hydrogel-based immobilization, emulsification, and nanoencapsulation enhance microbial survival against desiccation, UV radiation, and temperature extremes, ensuring that viable populations reach the rhizosphere (Riseh et al., 2023; Vassilev et al., 2020). Importantly, these systems facilitate synchronization between microbial activity and plant demand, a critical factor for maintaining soil biological balance in climate-stressed environments. However, challenges related to scalability, cost, and material optimization remain key barriers to large-scale deployment. Beyond formulation, the temporal stability and shelf life of PGPR products are crucial determinants of their practical applicability. Studies have demonstrated that optimized carrier systems can maintain high microbial viability over extended storage periods, ensuring consistent field performance and reliable soil health benefits (Parveen et al., 2023; Vassilev et al., 2020). Nevertheless, variability in environmental conditions during storage and application continues to influence microbial efficacy, highlighting the need for standardized protocols and robust formulation strategies.

### 8.2 Shelf life and stability of PGPR bioformulations

Shelf-life of PGPR formulations is important to achieve consistent biocontrol efficacy. Additives like starch, humic acid, skimmed milk, sugars, and nanomaterials increase shelf life and improve the overall efficacy of PGPR bioformulations in field conditions (Balla et al., 2022). Carrier materials such as alginate beads, talc and lignite were found to improve viability by 6–12 months and stabilization further improves pathogen suppression and field performance (Parveen et al., 2023). Increased stability prolongs the shelf life of PGPR inoculants and enhances effective colonization of the rhizosphere and enhanced plant growth-promoting efficacy under field conditions (Kumar et al., 2024). Shelf life of PGPR bioformulations is affected by carrier material, storage temperature, moisture and protective additives with appropriate formulations. Development of advanced formulations such as encapsulation, nano-biofertilizers, and biofilm based systems that improve microbial survival, stability and field performance (Monica et al., 2025). Encapsulated and nano-based liquid formulations generally provide more stability and longer shelf life than conventional carrier-based formulations by protecting PGPR from environmental stresses and ensuring gradual release. These sophisticated formulations improve the survival of the microbes, the quality of the biofertilizer and the performance in the field and the plant growth (Kanishka et al., 2025).

The shelf-life performance of *Pseudomonas fluorescens* and related biocontrol agents underscores their practical value in disease management. Arriel-Elias et al. (2018) reported that liquid formulations retained viable counts of approximately 10^8^ CFU/mL for up to 90 days when stored at 8 °C, with no loss in their ability to suppress *Fusarium* wilt in tomato and *Rhizoctonia solani* in rice even after three months of storage. Organic carriers appear to offer comparable, if not longer, protection: Bhattacharyya and Jha (2012) found that PGPR consortia comprising *Pseudomonas, Bacillus*, and *Azotobacter* species remained viable in peat and vermicompost carriers for 6–8 months, translating into a measurable reduction in soilborne disease incidence. Formulation chemistry also plays a role in extending viability; bioformulations supplemented with preservatives such as sorbate, glycerol, and molasses sustained unusually high cell densities (~21 × 10^8^ CFU/mL) over 12 weeks while retaining efficacy against damping-off in chili seedlings. Taken together, these findings suggest that storage stability is not merely a logistical concern but a functional determinant of biocontrol reliability: formulations that preserve viable cell counts over extended periods are consistently the ones that sustain disease-suppressive activity in the field.

## 9 AI-assisted bioformulation: predicting strain synergy and stability under drought stress

Artificial intelligence (AI)-assisted bioformulation is emerging as a state-of-the-art strategy for improving the reliability and field performance of plant growth-promoting rhizobacteria (PGPR), particularly under drought stress. Although PGPR are widely recognized for enhancing plant growth, nutrient acquisition, phytohormone production, osmotic adjustment, and stress resilience, their practical application is often limited by inconsistent survival, poor rhizosphere colonization, and variable compatibility among strains under fluctuating environmental conditions. Under drought, these limitations become even more critical, as reduced soil moisture, elevated temperature, and altered root exudation patterns can significantly impair microbial persistence and functionality. In this context, AI offers a promising framework for designing more robust, adaptive, and drought-resilient microbial formulations (Gupta et al., 2024; Mmotla et al., 2025). A major advancement in this area is the integration of multi-omics datasets, including metagenomics, transcriptomics, proteomics, and metabolomics with AI and machine learning algorithms to decode plant–microbe and microbe–microbe interactions. Bioinformatics platforms such as QIIME, MetaPhlAn2, MG-RAST, HUMAnN2, MetaTrans, MetaboAnalyst, MZMine, and XCMS enable the identification of functional traits associated with drought tolerance, such as ACC deaminase activity, exopolysaccharide (EPS) production, osmolyte synthesis, antioxidant induction, biofilm formation, and nutrient solubilization. (Al-Turki et al., 2023; Jain et al., 2024; Nadeem et al., 2021; Sharma et al., 2024). By integrating these datasets, AI can support the prediction of strain synergy and formulation compatibility, which is essential for developing effective microbial consortia. Instead of relying solely on conventional trial-and-error screening, machine learning models can identify cooperative strain combinations that maximize beneficial interactions while minimizing antagonism. Such models may also predict formulation stability, shelf-life, rhizosphere persistence, and environmental responsiveness, thereby improving inoculant performance under drought-prone field conditions (Gupta et al., 2024; Jain et al., 2024; Mmotla et al., 2025). Thus, AI-assisted bioformulation represents a significant step toward the development of precision microbial inoculants tailored for sustaining soil health and crop productivity under water-limited environments (Jain et al., 2024; Mmotla et al., 2025).

To integrate these diverse datasets, machine learning architectures such as random forest classifiers or artificial neural networks (ANNs) utilize the processed omics outputs as interconnected input features (Zhang et al., 2024). For example, an AI model can simultaneously analyze genomic abundance data from MG-RAST and metabolomic profiles from XCMS to uncover complex, non-linear correlations. This integration allows the algorithm to predict precisely how specific strains, such as *Pseudomonas putida*, might synergistically upregulate drought-tolerance traits like EPS production when co-cultured in a formulation, avoiding the need for exhaustive trial-and-error laboratory screening (Krantz et al., 2021).

### 9.1 IoT-based real-time monitoring: sensor networks for rhizosphere optimisation under drought stress

The Internet of Things (IoT) has emerged as a powerful tool for real-time rhizosphere monitoring and drought-responsive soil health management by enabling continuous sensing of the environmental variables that regulate both plant performance and PGPR functionality. Under drought conditions, soil health is not determined solely by water availability but also by the maintenance of microbial activity, nutrient mobility, soil aggregation, root-zone temperature, and biochemical stability. In PGPR-assisted systems, these factors are critical because drought can reduce microbial colonization, impair root exudation, and limit the expression of beneficial traits such as nutrient solubilisation, phytohormone production, and stress mitigation (Alzate Zuluaga et al., 2024; Chieb and Gachomo, 2023). State-of-the-art IoT platforms integrate soil moisture probes, temperature sensors, electrical conductivity (EC) meters, pH sensors, nutrient sensors, and microclimate monitoring units to generate high-resolution data from the rhizosphere (Abdelmoneim et al., 2025; Ndjuluwa et al., 2023).

These sensor networks enable the continuous tracking of dynamic root-zone conditions that influence microbial survival and soil biological function. Rather than relying on fixed irrigation schedules, IoT-enabled systems can support threshold-based irrigation and fertigation, allowing water and nutrient delivery to be adjusted according to soil moisture depletion, salinity build-up, and plant demand. This is particularly important for maintaining PGPR viability and activity in drought-prone soils, where excessive drying can reduce inoculant persistence and compromise biofilm formation and nutrient cycling (Abdelmoneim et al., 2025; Harkhani and Sharma, 2024; Pathmudi et al., 2023). Recent advances extend beyond simple sensing toward predictive and responsive soil management. Integration of IoT with cloud computing, remote sensing, and machine learning now allows early detection of drought stress through changes in canopy temperature, evapotranspiration, soil water imbalance, and root-zone environmental fluctuations (Abdelmoneim et al., 2025; Ali et al., 2024; Thingujam et al., 2025). Such systems are increasingly being used not only to conserve water, but also to preserve a microbe-supportive rhizosphere, thereby sustaining soil health and improving the consistency of PGPR-mediated drought resilience in precision agriculture (Ali et al., 2024; Alzate Zuluaga et al., 2024; Ndjuluwa et al., 2023).

When IoT sensor networks detect prolonged moisture deficits or thermal spikes that risk induced population decline or desiccation of the bioinoculants, the system can dynamically prompt targeted microbial reinoculation or trigger fertigation pulses containing protective carrier compounds. Aligning automated sensor feedback with microbial population thresholds ensures bioinoculants are replenished precisely when environmental conditions favor post application survival, directly tying irriagation and soil moisture metrics to precision PGPR management decisions (Ahmad Ansari et al., 2023). For example, during a severe mid-season heatwave, buried soil sensor might detect that moisture levels have dropped below critical thresholds and temperature have exceeded 35 °C for three consecutive days- condition know to impair bacterial viablility. Rather than merely scheduling standard irrigation, the automated platform recognize the compromised biological status of the applied plant growth- promoting rhizobacteria. It instantly initials a precise fertigation pulse carrying protective agent like trehalose, followed by the automated re-injection of fresh bioinoculant. Aligning sensor feedback with microbial theresholds ensures bioinculants are replenished precisely when envirnmnental conditions favor post application survival, directly tying soil metrics to precision PGPR management decisions (Pathmudi et al., 2023).

### 9.2 Smart-MIP (Microbial- and Sensor-Based Integrated Precision) frameworks

Smart-MIP (Microbial- and Sensor-Based Integrated Precision) frameworks have emerged as an advanced strategy for precision microbiome management through the integration of microbial technologies with sensor-based digital agriculture tools. These frameworks combine beneficial microbial inoculants, real-time environmental sensing, and AI-driven predictive analytics to support targeted, adaptive, and site-specific agricultural interventions (Abdelmoneim et al., 2025; Gupta et al., 2024). In the context of drought-resilient agriculture, Smart-MIP represents a shift from conventional input-based farming toward data-driven rhizosphere management, where both microbial performance and soil conditions are continuously optimized. Unlike traditional approaches that treat microbial application and environmental monitoring as separate processes, Smart-MIP frameworks integrate these components into a unified precision management platform. A practical example is the IoT-driven predictive management of *Bacillus*-based PGPR, where beneficial microbial populations can be indirectly monitored through sensor-derived environmental data. *Bacillus* species are particularly suitable for such systems because of their roles in phytohormone production, nutrient cycling, biofilm formation, and suppression of phytopathogens, all of which are important for maintaining plant and soil resilience under stress conditions (Chen et al., 2019; Lin et al., 2023). Conventional monitoring of beneficial microbes generally relies on periodic and labor-intensive laboratory analyses. In contrast, digital platforms such as AgriTalk integrate IoT-based soil sensing with machine learning models, including multilayer perceptrons, to predict *Bacillus* abundance in soil ecosystems. This demonstrates the operational basis of Smart-MIP, where microbial abundance and activity can be estimated using predictive sensor-based models rather than relying solely on destructive sampling. Conceptually, Smart-MIP functions as a multi-layered framework consisting of a microbial layer (selection and application of PGPR), a sensor layer (monitoring rhizosphere and environmental parameters), a data analytics layer (AI-based interpretation and prediction), and a precision application layer (site-specific interventions such as irrigation adjustment, nutrient dosing, or microbial reapplication) (Abdelmoneim et al., 2025; Jain et al., 2024; Lin et al., 2023; Sharma et al., 2024). Overall, Smart-MIP frameworks provide a promising platform for improving the consistency, scalability, and efficiency of PGPR application in sustainable and climate-resilient agriculture (Lin et al., 2023; Pathmudi et al., 2023).

### 9.3 IoT-based greenhouse gas monitoring for PGPR-assisted sustainable agriculture

PGPR such as, *Bacillus* and *Azospirillum spp*. offer a biological approach to increase crop yield and reduce agricultural greenhouse gas (GHG) emissions (Glick, 2012). PGPR reduce GHG fluxes by increasing the expression of nitrous oxide (N_2_O) reductase genes (nosZ) to minimize denitrification losses (Domeignoz-Horta et al., 2016), affecting methane (CH_4_) oxidation in flooded rhizosphere environments and promoting soil organic carbon (CO_2_) sequestration through root exudation (Vejan et al., 2016). However, the effectiveness of PGPR at the field scale is dynamic and varies with microclimatic variables. The discrete gas sampling by static chambers is usually not temporally resolved enough to adequately capture fast emission pulses or the continuous microbial kinetics (Butterbach-Bahl et al., 2013; Vejan et al., 2016).

Integrating Internet of Things (IoT) sensor networks resolves these temporal limitations by providing real-time flux tracking directly at the physical-chemical sensing interface (O'Grady and O'Hare, 2017). Solid-state Metal Oxide Semiconductor (MOS) sensors (SnO2/CuO) operating at detect and through chemiresistive electron transfer: reducing gases (CH_4_) release trapped electrons back to the conduction band to collapse the surface depletion barrier and decrease electrical resistance (ΔR), whereas oxidizing gases (N_2_O) trap additional electrons to increase resistance (Ji et al., 2019). Concurrently, Non-Dispersive Infrared (NDIR) sensors track concentrations via optical light attenuation.

Edge microcontrollers digitize these raw electrical and optical signals, compensate for ambient humidity drift using hydrophobic silanized membranes, and transmit payloads across Low-Power Wide-Area Networks (LoRaWAN or NB-IoT) to cloud platforms (Ji et al., 2019; Yang K. et al., 2026). At the cloud analytics level, machine learning models cross-reference real-time gas flux curves with co-located soil parameters (volumetric water content, temperature, redox potential, pH) to isolate specific PGPR metabolic pathways such as nosZ-driven suppression from background biological VOC releases (Tzounis et al., 2017). This integrated framework continuously verifies bio-based GHG mitigation, establishing automated triggers for PGPR reinoculation and closed-loop precision fertigation.

## 10 Conclusion

By regulating the soil-plant-microbe interface, PGPR facilitate landscape recovery through a complex hierarchy of molecular signaling and edaphic modifications. By altering root architecture, preserving hydraulic integrity, and controlling antioxidant activity, PGPR significantly increase plant resilience to abiotic stresses such as drought, salinity, heat, and flooding. Furthermore, by improving organic carbon sequestration and organizing soil architecture through the production of extracellular polymeric substances (EPS), these “rhizosphere architects” actively restore soil health. Additionally, PGPR drive the crucial bioremediation of terrestrial habitats by tolerating heavy metals, breaking down microplastics, and mineralizing persistent organic pollutants (POPs). However, presenting PGPR as a universal solution overlooks significant contradictory findings in the current literature. Field-scale applications frequently report inconsistent efficacy; inoculants that demonstrate high performance *in vitro* often fail to establish in native soils or, paradoxically, can suppress indigenous beneficial microbial populations. A major knowledge gap persists regarding the metabolic trade-offs and molecular cross-talk between introduced PGPR and native microbiomes, particularly under compounded abiotic stresses (e.g., simultaneous drought and heat). Furthermore, the long-term ecological consequences of repeatedly introducing engineered microbial consortia into fragile ecosystems remain largely undocumented. To address these gaps and bridge the “performance gap” between laboratory breakthroughs and field-scale application, future research must prioritize longitudinal, multi-environment field trials integrated with multi-omics approaches. The future of sustainable agriculture must also transition toward a digitally integrated Smart-MIP (Microbial- and Sensor-Based Integrated Precision) framework. These microbial “architects” can be precisely deployed by coordinating tailored bioformulations, such as biochar-encapsulated consortia, with AI-driven predictive strain selection and IoT-powered real-time rhizosphere monitoring. Ultimately, acknowledging these current limitations and pursuing this convergence of microbiology, material science, and digital intelligence provides a robust, empirically validated roadmap for climate-resilient farming and ecosystem restoration.
